# Directing isomerization reactions of cumulenes with electric fields

**DOI:** 10.1038/s41467-019-12487-w

**Published:** 2019-10-02

**Authors:** Yaping Zang, Qi Zou, Tianren Fu, Fay Ng, Brandon Fowler, Jingjing Yang, Hexing Li, Michael L. Steigerwald, Colin Nuckolls, Latha Venkataraman

**Affiliations:** 1 Department of Applied Physics, Columbia University, New York, New York USA; 2 Department of Chemistry, Columbia University, New York, New York USA; 3 Shanghai Key Laboratory of Materials Protection and Advanced Materials in Electric Power, Shanghai University of Electric Power, Shanghai, 200090 China

**Keywords:** Electrocatalysis, Molecular electronics

## Abstract

Electric fields have been proposed as having a distinct ability to catalyze chemical reactions through the stabilization of polar or ionic intermediate transition states. Although field-assisted catalysis is being researched, the ability to catalyze reactions in solution using electric fields remains elusive and the understanding of mechanisms of such catalysis is sparse. Here we show that an electric field can catalyze the cis-to-trans isomerization of [3]cumulene derivatives in solution, in a scanning tunneling microscope. We further show that the external electric field can alter the thermodynamics inhibiting the trans-to-cis reverse reaction, endowing the selectivity toward trans isomer. Using density functional theory-based calculations, we find that the applied electric field promotes a zwitterionic resonance form, which ensures a lower energy transition state for the isomerization reaction. The field also stabilizes the trans form, relative to the cis, dictating the cis/trans thermodynamics, driving the equilibrium product exclusively toward the trans.

## Abstract (web summary)

Electric fields have been proposed as having a distinct ability to catalyse chemical reactions through stabilizing polar intermediates. Here, the authors show that electric fields can catalyse the cis-to-trans isomerization reactions of cumulenes in solution in a scanning tunnelling microscope

## Introduction

Understanding and controlling electronic reorganizations in chemical reactions is at the heart of synthesis chemistry^1^. In principle, electric fields can catalyze chemical transformations through stabilizing otherwise unfavorable electronic structures, enabling new transition states, modifying reaction barriers^2–5^, and altering reaction selectivity^5–9^. For example, studies in electrochemical environments suggest that interfacial electric fields can be exploited to control the selectivity of non-faradaic reactions^10,11^, whereas vibrational Stark effect spectroscopy^4^ and single-crystal diffraction experiments^12^ have been utilized to measure the magnitude of the electric field at enzyme active sites and cocrystal systems. Although it is conceptually appealing and, in principle, widely applicable, the use of oriented electric fields to alter the electronic structure of molecular reactants and transition states is beset with challenges^13,14^. In this regards, the scanning tunneling microscopy (STM) offers an ideal platform with which to apply a large external electric field over the small distance between two electrodes to induce chemical transformations that can be probed directly by measuring single-molecule junction conductance. Although a previous STM-based study has demonstrated a field-induced single-molecule reaction, it was limited in scope due to an ultra-low conversion rate (a few molecules per hour)^2,15^.

The study based on STM-break junction (STM-BJ) technique described herein demonstrates that electric fields can direct and catalyze solution-phase reactions. We focus on [3]cumulene derivatives, which consists of chains of carbon atoms bound together to give contiguous π-bonds^16^, and follow the cis-to-trans isomerization reactions under an electric field. The experiments are performed using the STM-BJ technique^17,18^, which possesses the advantage of being able to apply a large (and possibly catalytic) electric field, while at the same time being able to detect (through measurements of single-molecular junction conductance) the chemical products of its catalysis. We also analyze the reactants and products formed within the STM setup using high-performance liquid chromatography (HPLC) and confirmed that the reaction indeed occurs in the solution phase. We show that, at room temperature, an electric field accelerates the otherwise extremely sluggish cis-to-trans isomerization of the cumulene. Density functional theory (DFT)-based calculations confirm that the electric field alters the [3]cumulene charge distribution promoting an ionic resonance form, thereby decreasing the isomerization barrier. The applied field also alters the reaction thermochemistry, favoring the trans form and determining the ultimate distribution of the products to one that cannot be achieved by simply heating or irradiating the solution.

## Results

### Synthesis of cumulenes

We design and synthesize an asymmetric [3]cumulene that has both an aurophilic (SMe) and an alkyl (t-Bu) substituent on each end. Each SMe terminal group binds to undercoordinated Au atoms on the STM tip and substrate, enabling single-molecule junction conductance measurements, whereas the t-Bu group provides steric bulk to prevent unwanted reactions of the cumulated double bonds to ensure air stability^19^. The synthesis, detailed in the Supplementary Methods, yields a mixture of the cis and trans isomers, **cis[3]** and **trans[3]**. We separated the isomers by fractional crystallization to isolate the pure **cis[3]** (23% yield) and **trans[3]** forms (28% yield), both of which are air stable as evidenced by 1H-nuclear magnetic resonance (NMR) experiments (see Supplementary Methods).

### Conductance measurements

**Fig. 1:**
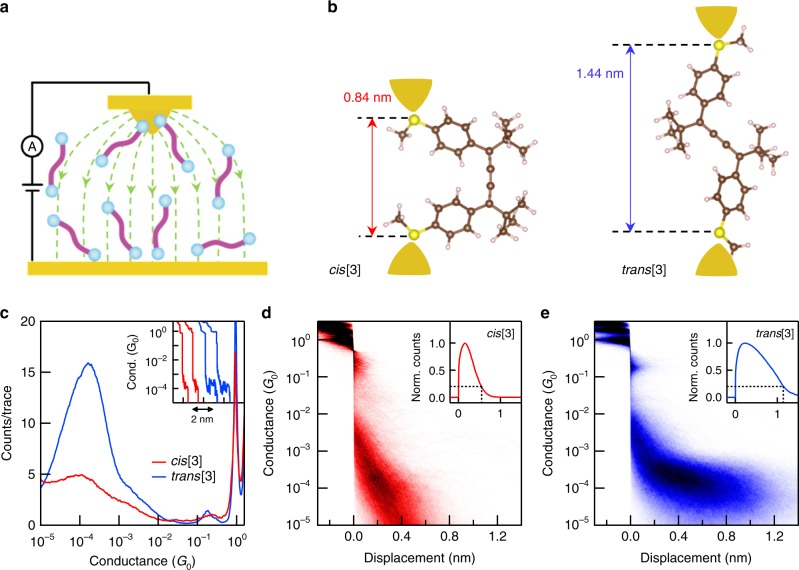
Conductance measurements of cumulene isomers in solution. **a** Schematic of the STM setup and the electric field distribution between the STM tip and substrate (green line). **b** Illustration of the single-molecule junctions with DFT-optimized structures of **cis[3]** and **trans[3]** showing distinct S–S distance. The electrodes are model structures not included in the calculations. **c** Logarithm-binned 1D histograms for **cis[3]** (red) and **trans[3]** (blue) measured in tetradecane at a 0.1 V bias created by compiling 5000 and 9000 conductance traces, respectively. Inset: example conductance vs. displacement traces of **cis[3]** (red) and **trans[3]** (blue). **d**, **e** 2D conductance-displacement histograms for **cis[3]** and **trans[3]**, respectively, created by overlaying conductance traces after aligning them at a conductance of 0.5 G_0_. Inset of **d** and **e**: normalized displacement profiles of **cis[3]** (red) and **trans[3]** (blue), respectively, determined from the 2D histograms. The 80th percentile junction length is 0.55 nm for **cis[3]** and 1.15 nm for **trans[3]** as indicated by the dashed lines

STM-BJ measurements are made using a custom apparatus^20^ that applies a large electric field to the molecular solution in the region between the STM tip (Fig. 1a) and substrate, and determines the conductance of single-molecule junctions (Fig. 1b)^18^. We describe the measurements, which are carried out at room temperature and in the dark, in the Methods section and Supplementary Note 1. In Fig. 1c, we present the single-molecule conductance data of **cis[3]** and **trans[3]** measured (separately) in tetradecane, a nonpolar solvent, at a tip bias of 0.1 V. The illustrative conductance traces (Fig. 1c inset) start with plateaus close to integer multiples of G_0_ ( = 2e^2^/h, conductance quantum) and show a molecular conductance plateau around 10^−4^ G_0_. Logarithmically binned one-dimensional (1D) conductance histograms of such traces, compiled without any data selection from thousands of traces, collected within a 2 h period are shown in the main panel. The peak around 10^−4^ G_0_ in the 1D histograms corresponds to the conductance of the single-molecule junctions. The peak for **cis[3]** is broad, whereas that for **trans[3]**, at a slightly higher conductance, is more well-defined. We attribute the similar conductance value of the two isomers to their similar electronic structure, as can be deduced from their electrochemical and optical characterizations and their DFT-based frontier orbitals (see Supplementary Figs. 1–3).

The distinct conformations for **cis[3]** and **trans[3]**, as determined by DFT-optimized molecular structures (see Methods) are shown in Fig. 1b with model electrodes to illustrate the length of a junction. The S–S distance of **cis[3]** and **trans[3]** differs by about 0.6 nm. This results in the conductance plateaus having different lengths as seen in the inset of Fig. 1c and in the two-dimensional (2D) conductance-displacement histograms (Fig. 1d, e). Length profiles of the 2D histogram features, shown in the inset of Fig. 1d, e, indicate that **trans[3]** junctions are roughly 0.6 nm longer than **cis[3]** ones. The experimental plateaus are shorter than the DFT-based junctions due to the gap that is formed after the Au-contact ruptures and relaxes^20^. Given the similar junction formation probability (>94%, see Supplementary Fig. 4 for the analysis), the longer junctions of **trans[3]** results in a stronger and more pronounced 1D histogram peak in contrast to that of the **cis[3]**. Taken together, these results illustrate the ability of the STM-BJ measurement to distinguish between **cis[3]** and **trans[3]** isomer-based molecular junctions.

### Isomerization reactions of cumulenes

**Fig. 2:**
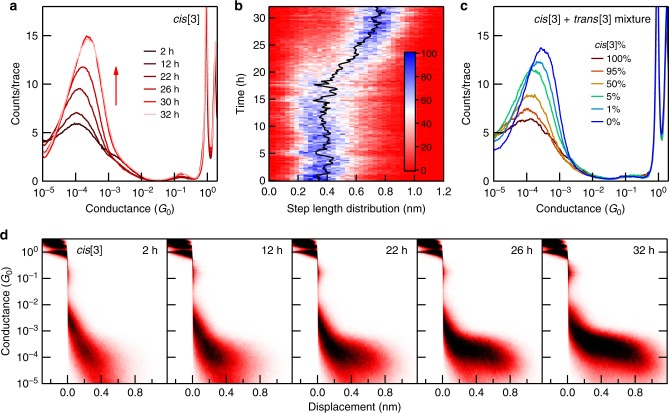
Isomerization reactions of cumulenes observed during conductance measurements. **a** Logarithmically binned 1D histograms of 10,000 consecutive conductance traces measured at different times as indicated for **cis[3]** in tetradecane at a bias of 0.1 V. **b** Conductance step length distributions as a function of time showing a clear transition from a predominantly shorter plateau at the start of the experiment to longer ones at the end. The image includes step length analysis of 155,000 conductance traces in sets of 1000 traces. The step length is determined by counting data points in each trace over a conductance range between 10^−3^ and 10^−4.9^ G_0_. The black line traces the peak position. **c** Logarithmically binned 1D histograms for different mixtures of **cis[3]** and **trans[3]** in tetradecane measured at a 0.1 V bias. Each histogram is compiled from 5000 conductance traces. **d** 2D conductance-displacement histograms compiled from the same **cis[3]** data shown in **a**

Focusing on the measurements of **cis[3]**, we find that the conductance histograms change over time. Figure 2a and Fig. 2d show 1D and 2D histograms compiled from 10,000 traces measured in ~2 h at different times over the 32 h period. The histogram peak heights and the conductance plateau lengths increase with time. After about 32 h, the conductance features are remarkably similar to those of **trans[3]** and do not change further. This change is also verified through conductance step length analysis of the 155,000 traces measured over the 32 h period (Fig. 2b). By contrast, a similar measurement of **trans[3]** lasting 60 h does not show any change (see Supplementary Figs. 5 and 6). We hypothesize that a selective cis-to-trans isomerization reaction takes place in our experimental setup.

To validate this hypothesis, we measure mixed solutions of **cis[3]** and **trans[3]** in which we vary their relative concentrations. The 1D and 2D histograms (generated by compiling thousands of traces collected in <2 h for each solution) show conductance peak heights and plateau lengths increase as the concentration of the trans isomer is increased (Fig. 2c and Supplementary Fig. 7). This result compares well with that of the pure **cis[3]** solution measured as a function of time (Fig. 2a, d), supporting the isomerization reaction from cis-to-trans. Comparing these data and examining the plateau length distribution of the **cis[3]** and **trans[3]** solutions, we determine that the 32 h measurement yields a solution that is >90% **trans[3]** (see Supplementary Fig. 8).

To exclude alternate explanations, we note first that all the measurements are performed in the dark; this rules out photoisomerization. Indeed, measurements in the presence of white light yield a ~ 40:60 mixture of cis:trans, as determined by 1H-NMR and STM-BJ characterizations shown in Supplementary Figs. 9 and 10. Using a combination of variable-temperature 1H-NMR and UV-vis spectroscopy, we find that a thermal equilibrium between cis and trans is achieved at 410 K; this implies a barrier to isomerization likely larger than 1 eV (Supplementary Figs. 11 and 12)^21,22^. Importantly, at room temperature, no isomerization is seen in over 72 h (Supplementary Fig. 2). We also examined each of the two isomers in solution with gold nanoparticles using variable-temperature 1H-NMR characterizations (Supplementary Figs. 13 and 14) and we found that the nanoparticles do not affect the dynamics of the isomerization; thus, we exclude the possibility of the isomerization being simply gold-catalyzed. We deduce that the reaction is catalyzed during the STM-BJ process.

The mixed-solution STM results (Fig. 2c) show clearly that we convert not just the fraction of molecules that form molecular junctions but close to the entire solution used in the STM measurements. As we repeat the break-junction measurement 155,000 times over the 32 h period with a solution that has ~10^15^ molecules, the isomerization reaction cannot be occurring only to the small fraction of molecules that are held between the STM tip and substrate. This rules out mechanisms such as those induced by electron tunneling excitations, resonant transport (see Supplementary Fig. 15), or mechanical forces in the junction^23–27^. Finally, we repeat the STM-BJ measurement for a larger quantity of the **cis[3]** solution for ~240 h, extract this solution from the STM setup, and analyze it using HPLC. We find that the cis:trans ratio for this solution is 30:70 compared with a ratio of 92:8 for a control solution left out for the same amount of time under environmental light (Supplementary Fig. 16). Significantly, the factor of ~27 increase in the relative ratios demonstrates conclusively that the solution extracted from the STM setup is converted selectively from pure cis to predominantly trans.

**Fig. 3:**
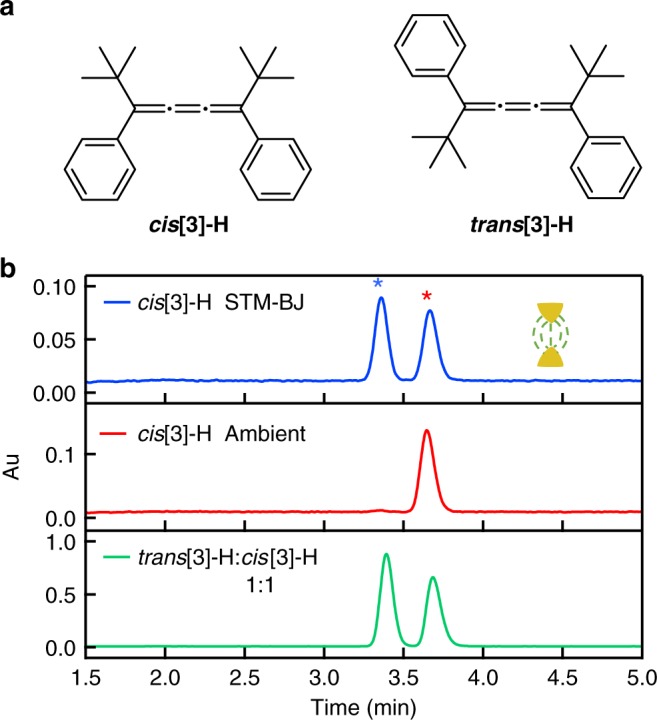
Isomerization reactions of cumulenes without aurophilic linkers. **a** Molecular structure of **cis[3]-H** and **trans[3]-H** cumulene isomers. **b** HPLC spectra for **cis[3]-H** collected after STM-BJ measurements of ~300 h in TD at 0.5 V (blue); the same solution kept outside the setup for the same amount of time (red); a 1:1 mixture of **cis[3]-H** and **trans[3]-H** (green). The signature peak of **cis[3]-H** and **trans[3]-H** is denoted by the red and blue stars, respectively

To illustrate the generality of these results, we turn to an additional control experiment performed using the same [3]cumulene backbone but without any SMe linkers. The molecules, **cis[3]-H** and **trans[3]-H** (Fig. 3a), do not form molecular junctions, as they have no gold-binding linkers. We synthesize these molecules as detailed in Supplementary Methods and carry out STM-BJ measurements (repeatedly forming and breaking gold-point contacts) in the dark with a 0.3 ml solution of **cis[3]-H** for over 300 h, while applying a 0.5 V bias. Conductance histograms do not show any clear molecular peaks (Supplementary Fig. 17). However, an HPLC analysis of the solution after the measurement (see Methods) shows that we have created a mixture with a **trans[3]-H** to **cis[3]-H** ratio that is roughly 1:1 (Fig. 3b). Comparing this HPLC spectrum with that of the same **cis[3]-H** solution kept outside the STM-BJ in dark for the same 300 h demonstrates indisputably that the isomerization reaction is catalyzed during the STM-BJ process. These results further confirm that the isomerization reaction is a solution-phase process where the reactants do not need to form molecular junctions.

STM-BJ measurements involve repeatedly forming and breaking a gold-point contact, while applying a bias voltage. The time-averaged separation between the tip and substrate is roughly 1 nm; therefore, the solution around the tip/substrate volume is continuously exposed to a high electric field. This field can affect all of the molecules distributed around the electrode gap and over time (owing to the diffusion of the molecules in solution and local turbulence created by the movement of the tip) impact the entire sample. We deduce that the observed isomerization reactions are catalyzed by an electric field. To test whether this is indeed the case, we carry out control experiments with the SMe-terminated **cis[3]** compound. We collected 1000 conductance traces and then either turn off the bias or keep the STM tip retracted by ~1 µm from the substrate with 0.1 V bias applied for a 2 h period and repeated the cycle for over 36 h. Conductance histograms from these measurements show no conversion from **cis[3]** to **trans[3]** (see Supplementary Fig. 18). This therefore confirms that an electric field is necessary to drive the isomerization reaction.

As the electric field distribution depends on the polarity of the solvent, we carry out additional control measurements of **cis[3]** in different solvents including 1,2,4-trichlorobenzene (TCB, a nonpolar solvent) and propylene carbonate (PC, a polar solvent) with a supporting electrolyte (tetrabutylammonium perchlorate), which screens the field. In TCB, again, we observed a cis-to-trans conversion, which takes ~20 h (Supplementary Fig. 19). By contrast, there is no conversion in PC even after 50 h (Supplementary Fig. 20). The control measurement in PC additionally rules out an electrocatalytic mechanism where the reaction is induced by charge transfer at the electrode surface^28^.

### Theoretical analysis

**Fig. 4:**
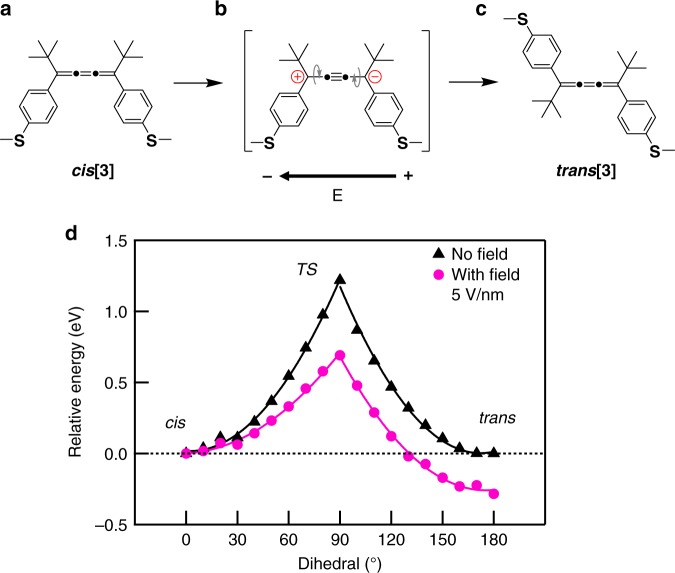
Calculated reaction energy with and without field. **a** Standard canonical resonance structure and **b** zwitterionic resonance structure of **cis[3]**. **c** Standard canonical resonance structure of **trans[3]**. Gray arrows illustrate the rotation along the C–C bonds. **d** Relative energy calculated without (black) and with an electric field of 5 V/nm (magenta). Note that the curves are offset vertically to set the energy of cis to 0

To understand how the electric field can catalyze the reaction, we first point out that the cumulenes have many π-bonds^29,30^. Rather than the standard canonical resonance structure of **cis[3]** (Fig. 4a), under an applied electric field, the polar zwitterionic resonance structure (Fig. 4b) is enhanced. The important feature to the zwitterionic resonance structure is the (relatively) free rotation around the terminal C–C bonds, as the terminal π-bonds are absent. Thus, an electrostatic polarization, which promotes the zwitterionic resonance structure, can reduce the rotational barrier making isomerization energetically accessible (see Supplementary Note 2 and Supplementary Fig. 21 for more discussions). In addition, the applied field dictates the ultimate product distribution, as **trans[3]** has a larger field-induced dipole moment than **cis[3]** (see Supplementary Fig. 22). Therefore, there is a driving force for the conversion of **cis[3]** to **trans[3]** but not the reverse.

To explore this hypothesis more quantitatively, we carry out DFT calculations and explore the energy costs for the isomerization both with and without an electric field. Calculation details are provided in the Methods section and Supplementary Note 2. We find that the barrier for interconversion between **cis[3]** (dihedral of 0°) and **trans[3]** (dihedral of 180°) is >1 eV (Fig. 4b) in the absence of an electric field. This agrees with the variable-temperature 1 H NMR characterizations presented in Supplementary Figs. 11 and 12. Analyzing the structure (see Supplementary Note 2 for details), we find that the transition state (90° dihedral angle) has a larger dipole moment than either the cis or the trans form (see Supplementary Fig. 22). This electronic rearrangement in going from either the cis or trans form to the 90° twisted form accounts, in part, for the large activation energy required for the interconversion. We note that the cis and trans forms are isoenergetic in the absence of a field.

With an electric field of 5 V/nm applied along the cumulene backbone, we find that the dipole moment increases in both the cis and trans form as the field stabilizes a charge-separated resonant form (see Supplementary Fig. 22). This electronic reorganization facilitates the isomerization, as the more ionic transition state (90° dihedral) is stabilized more than the **cis[3]** reactant by the field. Furthermore, these calculations show that **trans[3]**, which has a much larger dipole moment than **cis[3]** (28.8 D vs. 19.3 D), has a lower energy in a field. This can be attributed to a more efficient charge delocalization over the longer length of the trans isomer compared with the cis. These effects result in a lower activation barrier for isomerization, which favors the trans product in an electric field (Fig. 4d), while also impeding the reverse reaction to cis.

To better evaluate the catalytic strength induced by external field, we calculate the relative cis-to-trans reaction rate under different field strengths. As shown in Supplementary Fig. 23, a field of 1 V/nm increase the cis-to-trans reaction rate by a factor of 6. When the field strength is increased to 5 V/nm, the reaction is accelerated by a factor of 109. This suggests that the electric field is a powerful catalyst for speeding up isomerization. Moreover, we compare these field-induced dipole moments with the permanent dipole of a series of analogous molecules with donor and acceptor substitutions on the two ends. The polarization induced by external field is comparable to that of the molecule with charged functional groups (see Supplementary Fig. 24). The transformation from electrostatic strength to chemical strength provides insights for designing molecular electrostatic catalytic reactions by employing internal field created by chemically engineering molecular polarization.

We note that the fields used in the calculations are considerably larger than what would be expected considering a simple parallel plate capacitor with a separation of ~1 nm under a 0.1 V bias for the tip-substrate pair. However, in STM-BJ measurements, the tip is repeatedly brought in and out of contact with the substrate at room temperature. Because of the electrode movements and gold atom reorganization, the distance between electrodes and therefore the field strength is changing during the measurements. The exact electric field around the apex of an atomically sharp STM tip is likely to be larger.

## Discussion

This study demonstrates unambiguously that electric field catalysis can be achieved in a solution environment using STM techniques where large catalytic electric fields can be created between the STM tip and substrate, while also enabling the detection of the reactants/products in situ through single-molecule conductance measurements. We show that an external electric field can increase the isomerization reaction rate and while also altering its selectivity. Through DFT calculations, we show that the reaction kinetics and thermodynamics are altered by the electrostatic field. The isomerization reaction of the cumulenes that have no aurophilic linkers further demonstrates that the external applied field aligns molecules along the reaction axis, facilitating scalable applications of solution-based electric field-driven catalysis. Our experiments however do not allow us to determine, quantitatively, field-dependent reaction rates, as we cannot determine the magnitude of the field between the STM tip and substrate in the solution environment. Moreover, in contrast with conventional chemical synthesis (which are typically characterized via HPLC or NMR techniques), nanoscale techniques cannot be applied for large-scale catalysis.

## Methods

### STM-BJ measurements

We repeatedly form and break point contact between a gold STM tip and substrate in a dilute solution (0.1 mM) of the target molecule under ambient conditions at room temperature in a custom setup^31^. After the Au-point contact is broken, a molecule bridges the gap between the gold electrodes to form a single-molecule junction. Conductance is measured as a function of the tip-substrate displacement under an applied bias voltage, yielding traces that reveal molecular-dependent conductance plateaus. With these measurements, we obtain molecular junction-specific conductance by compiling measured traces into 1D conductance histograms and the molecular backbone length from 2D conductance-displacement histograms.

### HPLC measurements

All samples were resolved by an Agilent 1200 Series analytical HPLC equipped with a diode array detector (220–900 nm) on a CHIRALPAK IA-3 column (4.6 mm ID × 250 mm, 3 μm) from Chiral Technologies, premixed 0.5% CH_2_Cl_2_/hexanes as the mobile phase, 1.00 mL/min flow rate, detection *λ* = 350 nm.

### DFT calculations

The geometry optimization and calculation of molecular total energy was carried out using the Fritz Haber Institute ab initio molecular simulation (FHI-aims) package^32,33^, using DFT with generalized gradient approximations for exchange-correlations energy developed by Becke, 3-parameter, Lee-Yang-Parr. The calculations are performed without and with an electric field by determining the energy for optimized structures at a range of dihedral angles.

## Supplementary information

Supplementary Information

## Data Availability

The data that support the findings of this study not included in the supplementary information document are available from the corresponding author upon reasonable request.

## Code availability

The data that support the findings was acquired using a custom instrument controlled by custom software (Igor Pro, Wavemetrics). The software is available from the corresponding author upon reasonable request.
