# Supplementary material for: Directing isomerization reactions of cumulenes with electric fields
Source: Nat Commun. 2019 Oct 2;10:4482. doi: 10.1038/s41467-019-12487-w (PMC6775130; doi:10.1038/s41467-019-12487-w)
Supplement: Supplementary file 1 — Supplementary Information [file 41467_2019_12487_MOESM1_ESM.pdf]

Supplementary Information

Directing isomerization reactions of cumulenes with electric Fields

Zang et al.

Supplementary Methods.

All reactions were performed in oven-dried or flame-dried round bottom flasks, unless otherwise noted. The flasks were fitted with Teflon magnetic stir bar, rubber septa and reactions were conducted under a positive pressure of nitrogen, unless otherwise noted. Anhydrous and anaerobic solvents were obtained from Schlenk manifold with purification columns packed with activated alumina and supported copper catalyst (Glass Contour, Irvine, CA). Automated flash chromatography was performed using a Teledyne Isco Combiflash R_f200 and Redisep R_f Silica/Alumina columns.

Materials. All chemicals were purchased from commercial sources and used without further purification unless otherwise specified.

Instrumentation. ^{1}H NMR and ^{13}C NMR spectra in deuterated solvents were recorded on Brucker DRX400 (400 MHz) or a Bruker DMX500 (500 MHz) spectrometer. NMR spectra are available in Supplementary Figures 25-45. Chemical shifts for protons are reported in parts per million (ppm) downfield from tetramethylsilane and are referenced to residual protium in the NMR solvents ($CHCl_3$: δ 7.26; $C_2H_2Cl_4$: δ 6.00; benzene: δ 7.16; CH_2Cl_2: δ 5.32). Chemical shifts for carbon are reported in parts per million downfield from tetramethylsilane and are referenced to the carbon resonances of the solvent ($CHCl_3$: δ 77.0; CH_2Cl_2: δ 53.84). Data are represented as follows: chemical shift, multiplicity (s = singlet, d = doublet, m = multiplet, br = broad), coupling constants in Hertz, and integration. High-resolution mass spectrometry (HRMS) were recorded on a Waters XEVO G2-XS QTOF spectrometer with dichloromethane as solvent. UV−vis absorption spectra were recorded on a Shimadzu 1800 spectrophotometer; spectrophotometer was standardized. Single crystal data was collected on an Agilent SuperNova diffractometer using a mirror-monochromated Mo or Cu Kα radiation.

X-ray crystallography. The crystal was grew by slowly diffusing ethanol into the solution of target molecule in hexane. The rod-shaped crystal was picked up from solution and mounted on a MiTeGen Kapton loop (polyimide). These were cooled to 100 K with an Oxford Cryosystems nitrogen flow apparatus. Data integration, scaling (ABSPACK) and absorption correction were perfomed in CrysAlisPro.2. Structure solution was performed using ShelXT.[1] Subsequent refinement was performed by full-matrix least-squares on F^2 in ShelXL. Olex2[2] was used for viewing and to prepare CIF files. Details of crystallographic data and refinement parameters are given in Supplementary Tables 1-2.

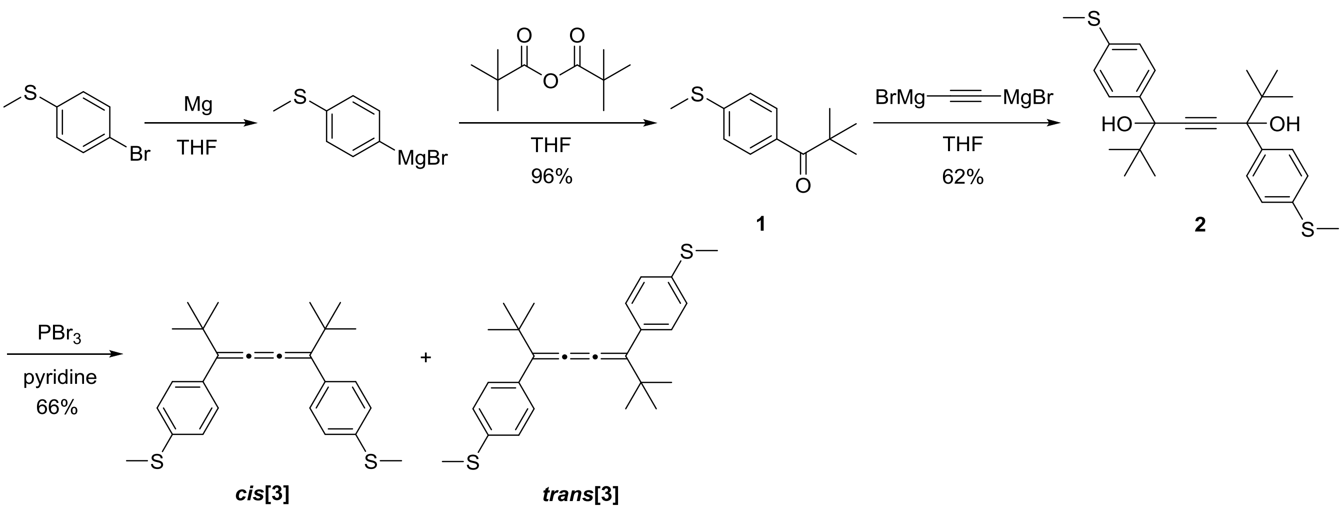

Synthesis of 2,2-dimethyl-1-(4-(methylthio)phenyl)propan-1-one (1): Under nitrogen atmosphere, a mixture of magnesium (2.67 g, 0.11 mol) and a pinch of iodine followed by anhydrous THF (40.0 mL) were added to the flask. The resultant suspension was heated to 30 ℃, subsequently a small amount of 4-bromothioanisole (20.3 g, 0.10 mol) in anhydrous THF (40.0 mL) solution was added to initiate the reaction until the brown color faded. Then the remaining solution was added dropwise over a period of an hour to maintain the temperature between 25 ℃ to 30 ℃. The reaction was continued for an additional 1 h at 25 ℃. The reaction mixture was transferred to a solution of trimethylacetic anhydride (22.4 g, 0.12 mol) in anhydrous THF (20.0 mL) via cannula at -30 ℃. After additional stirring for 6 hours at ambient temperature, the mixture was poured into icy 1 M HCl aqueous solution (100.0 mL) and then extracted with ethyl acetate (50.0 mL×3). The combined organic extracts were washed with saturated $NaHCO_3$ solution (50.0 mL) and dried over anhydrous Na_2SO_4, filtered, and concentrated. The residue was purified by distillation to give the compound **1** (20.0 g, 96%) as a colorless liquid. **^{1}H NMR** (400 MHz, $CDCl_3$): δ 7.72 (d, *J* = 8.0 Hz, 2H, benzene-H), 7.23 (d, *J* = 8.0 Hz, 2H, benzene-H), 2.51 (s, 3H, $-SCH_3$), 1.36 (s, 9H, $-C(CH_3)_3$); **^{13}C NMR** (100 MHz, $CDCl_3$): δ 207.03, 143.32, 133.94, 128.98, 124.83, 43.98, 28.20, 14.88; **HRMS (ASAP)**: $[M+H]^+$ calcd. for $C_{12}H_{17}OS$, m/z: 209.1000; found, 209.1020.

Synthesis of 2,2,7,7-tetramethyl-3,6-bis(4-(methylthio)phenyl)oct-4-yne-3,6-diol (2): Under nitrogen atmosphere, a solution of ethylmagnesium bromide (1.83 mL, 5.50 mmol, 3.0 M in Et_2O) in anhydrous THF (18.0 mL) was introduced to ethynylmagnesium bromide (10.0 mL, 5.0 mmol, 0.5 M in THF) via a dropping funnel over a 30 min period, with stirring. After completing the addition, the reaction mixture was refluxed for 2 h to give bis(bromomagnesium)acetylene. After cooling to the room temperature, the solution of compound **1** (2.08 g, 10.0 mmol) in anhydrous THF (5.0 mL) was added dropwise over a 10

min period. Then the mixture was refluxed for 3 h. After cooling to room temperature, the solution was poured into icy 1 M HCl aqueous solution (50.0 mL) and extracted with ethyl acetate (30.0 mL×3). The combined organic layers were washed with saturated $NaHCO_3$ solution (30.0 mL) and dried over anhydrous Na_2SO_4, filtered, and concentrated. The residue was purified by column chromatography (40 g Redisep R_f Silica) using a gradient from 0% to 30% ethyl acetate/hexanes to give the compound **2** (1.38 g, 62%) as a white solid. **^{1}H NMR** (500 MHz, $CDCl_3$): δ 7.49-7.52 (m, 4H, benzene-H), 7.18-7.21 (m, 4H, benzene-H), 2.49 (d, 6H, $-SCH_3$), 2.26 (d, 2H, -OH), 1.04 (s, 18H, $-C(CH_3)_3$); **^{13}C NMR** (125 MHz, $CDCl_3$): δ 138.99, 138.93, 137.63, 128.10, 128.07, 125.11, 88.79, 88.77, 78.85, 78.83, 39.79, 39.77, 25.53, 15.70, 15.68; **HRMS (ASAP)**: $[M-OH]^+$ calcd. for $C_{26}H_{33}OS_2$, m/z: 425.1973; found, 425.1976.

Synthesis of *cis*[3] and *trans*[3]: Under nitrogen atmosphere, to the solution of compound **2** (0.881 g, 1.99 mmol) in anhydrous pyridine (17.5 mL), phosphorus tribromide (0.6 mL, 6 mmol) was added dropwise over a 5 min period at room temperature and stirred for another 1 h. After cooling to –30 ℃, water (30.0 mL) was added. The resulting yellow mixture was extracted with dichloromethane (30.0 mL ×3), and the combined organic layers were washed with saturated $NaHCO_3$ solution (20.0 mL) and dried over anhydrous Na_2SO_4, filtered, and concentrated. The residue was purified by column chromatography (80 g Redisep R_f alumina) using hexanes to give the mixture of ***trans*[3]** and ***cis*[3]** (0.537 g, 66%) as a yellow solid. The mixture was recrystallized with ethyl acetate and hexanes to yield ***trans*[3]** (0.225 g) as a yellow-green needle crystal in residue, the filtrate was concentrated and recrystallized again with ethyl acetate and hexanes to yield ***cis*[3]** (0.190 g) as a pale-yellow plate-like solid in the filtrate.

For ***cis*[3]**, **^{1}H NMR** (400 MHz, $CDCl_3$): δ 7.30 (d, *J* = 12.0 Hz, 4H, benzene-H), 7.13 (d, *J* = 8.0 Hz, 4H, benzene-H), 2.48 (s, 6H, $-SCH_3$), 1.35 (s, 18H, $-C(CH_3)_3$); **^{13}C NMR** (125 MHz, $CDCl_3$): δ 156.77, 136.81, 136.67, 129.34, 129.25, 125.70, 37.40, 30.95, 15.71; **HRMS (ASAP)**: $[M+H]^+$ calcd. for $C_{26}H_{33}S_2$, m/z: 409.2024; found, 409.2024.

For ***trans*[3]**, **^{1}H NMR** (400 MHz, $CDCl_3$): δ 7.41 (d, *J* = 8.0 Hz, 4H, benzene-H), 7.20 (d, *J* = 12.0 Hz, 4H, benzene-H), 2.50 (s, 6H, $-SCH_3$), 1.25 (s, 18H, $-C(CH_3)_3$); **^{13}C NMR** (125 MHz, $CDCl_3$): δ 157.05, 136.78, 136.76, 129.22, 129.12, 125.72, 37.49, 30.72, 15.69; **HRMS (ASAP)**: $[M+H]^+$ calcd. for $C_{26}H_{33}S_2$, m/z: 409.2024; found, 409.2012.

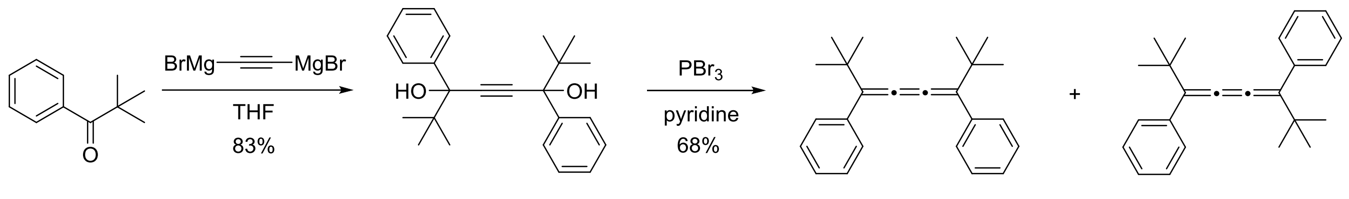

Synthesis of [3]cumulene derivatives *cis*[3]-H and *trans*[3]-H. Synthesis of 2,2,7,7-tetramethyl-3,6-diphenyloct-4-yne-3,6-diol (3) Under nitrogen atmosphere, ethylmagnesium bromide (2.2 mL, 6.6 mmol, 3.0 M in Et_2O) was introduced to a solution of ethynylmagnesium bromide (12.0 mL, 6.0 mmol, 0.5 M in THF) in THF (12.5 mL) via a dropping funnel over a 10 min period at room temperature, with stirring. After completing the addition, the reaction mixture was refluxed for 2 h to give a product which was white bis(bromomagnesium)acetylene. After cooling to the room temperature, the solution of 2,2-dimethylpropiophenone (1.98 g, 12.0 mmol) in THF (1.0 mL) was added dropwise over a 10 min period. Then the mixture was refluxed for 4.5 h. After cooling to room temperature, the solution was poured into icy 1 M HCl aqueous solution (80.0 mL), extracted with ethyl acetate (40.0 mL×3), combined organic layers were washed with saturated $NaHCO_3$ solution (40.0 mL) and dried over anhydrous Na_2SO_4, filtered, and concentrated. The residue was purified by column chromatography (40 g Redisep R_f Silica) using a gradient from 0% to 20% ethyl acetate/hexanes to give the compound **3** (1.75 g, 83%) as an off-white viscous solid. ^1H NMR (400 MHz, $CDCl_3$) δ (ppm): 1.06 (s, 18H, -$C(CH_3)_3$), 2.27 (br, 2H, -OH), 7.27-7.35 (m, 6H, benzene-H), 7.60-7.63 (m, 4H, benzene-H). ^{13}C NMR (100 MHz, $CDCl_3$) δ (ppm): 25.60, 39.70, 39.73, 79.11, 88.88, 88.92, 127.11, 127.41, 127.63, 127.65, 142.08, 142.14. HRMS (ASAP+, m/z): $[M+H]^+$ calcd for $C_{24}H_{31}O_2$, 351.2324; found, 351.2318.

Synthesis of *cis*[3]-H and *trans*[3]-H. Under nitrogen atmosphere, to the solution of compound **3** (0.21 g, 0.60 mmol) in anhydrous pyridine (4.8 mL), phosphorus tribromide (0.17 mL, 1.8 mmol) was added dropwise over a 5 min period at room temperature and stirred for another 1 h. Then the resulting mixture was heated to 90 ℃ and stirred at this temperature for 1.5 h. After cooling to -30 ℃, water (30.0 mL) was added. The resulting light-yellow mixture was extracted with ethyl acetate (30.0 mL×3), combined organic layers were washed with saturated $NaHCO_3$ solution (30.0 mL) and dried over anhydrous Na_2SO_4, filtered, and concentrated. The residue was purified by column chromatography (24 g Redisep R_f alumina) using hexanes to give the mixture product (0.129 g, 68%) as a light-yellow solid. The mixture was purified

on silica TLC plate (2000 μM, 60 Å) with hexanes as eluent to yield ***cis*[3]-H** (0.057 g) as a white solid with high polarity and ***trans*[3]-H** (0.065 g) as a white solid with low polarity, respectively.

For ***cis*[3]-H**, **^{1}H NMR** (400 MHz, CD_2Cl_2): δ 7.30-7.33 (m, 4H, benzene-H), 7.17-7.26 (m, 6H, benzene-H), 1.33 (s, 18H, -$C(CH_3)_3$); **^{13}C NMR** (100 MHz, CD_2Cl_2): δ 157.55, 140.10, 129.90, 128.79, 127.70, 126.63, 37.42, 30.51; **HRMS (ASAP)**: $[M+H]^+$ calcd. for $C_{24}H_{29}$, m/z: 317.2269; found, 317.2262.

For ***trans*[3]-H**, **^{1}H NMR** (400 MHz, CD_2Cl_2): δ 7.45-7.47 (m, 4H, benzene-H), 7.30-7.34 (m, 4H, benzene-H), 7.23-7.27 (m, 2H, benzene-H), 1.23 (s, 18H, -$C(CH_3)_3$); ^{13}C NMR (100 MHz, CD_2Cl_2): δ 158.15, 140.42, 130.24, 129.10, 128.16, 127.15, 37.80, 30.76; **HRMS (ASAP)**: $[M+H]^+$ calcd. for $C_{24}H_{29}$, m/z: 317.2269; found, 317.2267.

Supplementary Note 1. STM-BJ Method.

Conductance measurements were made using a custom scanning tunneling microscope (STM) that has been described in detail before.[3] Briefly, we use a mechanically cut 0.25 mm gold wire (99.998%, Alfa Aesar) as the STM tip and a gold-coated (99.999%, Alfa Aesar) steel substrate. A commercially available z-axis piezoelectric positioner (Nano-P15, Mad City Labs) is used to drive the tip in and out of contact with the substrate at a speed of 20 nm/s in a dilute solution of the target molecule in ambient environment at room temperature. The junction current (I) is recorded as a function of tip-substrate displacement at a fixed applied bias voltage (with a 100 kΩ resistor in series). The current, voltage across the junction and electrode position data are collected at a 40 kHz acquisition rate using custom Igor Pro (Wavemetric, Inc.) Conductance (G=I/V) is determined as a function of displacement and analyzed further using 1D and 2D histograms. Junctions start with a conductance of at least 5 G_0 (where G_0 is conductance quantum). Once the Au-point contact is broken, a molecule bridges the gap between the gold electrodes to form a single-molecule junction. By further withdrawing the tip, the junction breaks, causing a conductance drop to the instrumental noise floor (typically below 10^{-5} G_0 with a 0.1V bias). All conductance traces are compiled into conductance histograms without any data selection. We construct one-dimensional conductance histograms using logarithmic bins (100/decade), and two-dimensional histograms use logarithmic bins along the conductance axis (100/decade) and linear bins (1250/nm) along the displacement axis.

Standard conductance measurements. Conductance measurements of cumulene molecules were carried out in dilute solutions (~0.1 mM). For measurements in polar solvents, the STM tip was coated with Apiezon wax to decrease ionic currents.[4] All the measurements are performed using fresh solutions, without any exposure to light, and in a dark acoustic box unless otherwise specified. To study the effect of the electric field on these measurements, we collected over hundred thousand traces using the same tip/sample pair and solution. Time dependent histograms were created by compiling 10000 traces measured over a ~2 hour period and repeating this analysis periodically over the time scale of the measurement.

Control measurements. 1. We collected 1000 traces in a fresh ***cis*[3]** TD solution and then retract the tip by about ~1 μm, waited for 2 hours and repeated this cycle over a period of 36 hours. All measured traces were compiled into 1D and 2D histograms without any data selection (see Supplementary Figure 17). 2. We collected 1000 traces in a fresh ***cis*[3]** solution in TD and then turned off the bias, waited for 2 hours and repeated this cycle over a period of 44 hours. All measured traces were compiled into 1D and 2D

histograms without any data selection (see Supplementary Figure 17). 3. We performed the standard conductance measurements with a fresh ***cis*[3]** solution in TD after exposure to white light illumination for several hours. To present the time dependence of the histograms, we compiled 5000 traces measured over a ~1 hr period and repeated this process periodically over the time scale of the experiment.

Supplementary Note 2. DFT Calculations.

The geometry optimization and calculation of molecular total energy was carried out using the Fritz Haber Institute *ab initio* molecular simulation (FHI-aims) package[5,6], using density functional theory (DFT) with generalized gradient approximations for exchange-correlations energy developed by Becke, 3-parameter, Lee-Yang-Parr (B3LYP). These calculations are performed without considering solvents. The molecular geometry was relaxed and optimized until all force components on every atom was smaller than 10^{-2} eV/Å. For constrained relaxation that maintained a fixed dihedral angle, the coordinates of C1, C4, C5 and C6 were confined (see Supplementary Figure 22) while allowing all the other atoms remain free. A dihedral of 0 °corresponds to the *cis* isomer and a dihedral of 180 °corresponds to the *trans* isomer. For simulation under field, an external homogeneous electrical field is applied. Relaxation under the field starts from an optimized structure without field and is carried out iteratively. During each iteration, the direction of field is rotated to minimize the total energy under field, and then the molecule geometry is optimized maintaining the field orientation. Iterations are continued until the change in energy is smaller than 1×10^{-4} eV. Supplementary Figure 22b presents the dipole moments of ***cis*[3]**, ***trans*[3]** and the transition state conformer under an oriented electric field of 5 V/nm. For ***cis*[3]**, ***trans*[3]** and intermediates with dihedral 60°, 90° and 120°, the molecular energy is calculated under electrical field of different intensity between 0 to 5 V/nm. Due to the dipole moment and polarizability of molecule, the energy is lowered under field after a free rotation and relaxation. As dihedral angle, starting with ***cis*[3]** rotates to form ***trans*[3]**, the energy of molecule reaches its maximum at 90°. Hence, we can regard the energy difference between 90° and 0° as the activation energy of reaction ($\Delta H^{\neq}$). As the applied external electrical field increases, the $\Delta H^{\neq}$ decreases, as shown in Supplementary Figure 23. This rationalizes our finding that the field moves the equilibrium to the *trans* isomer and accelerates the isomerization.

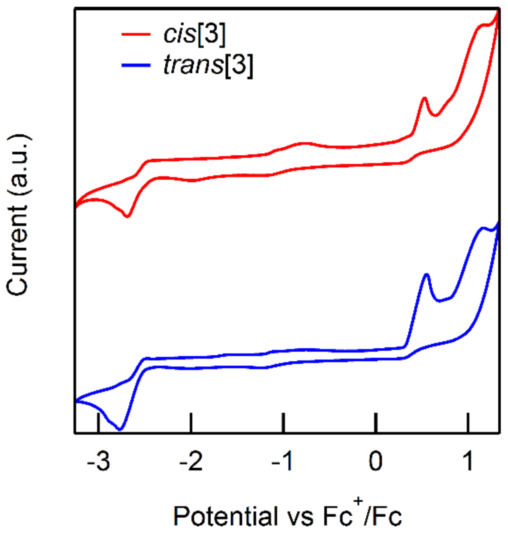

Supplementary Figure 1. Electrochemical characterizations. Cyclic voltammograms of ***cis*[3]** and ***trans*[3]** in 0.1 M $TBAPF_6$ in THF with a 100 mV/s scan rate. ***cis*[3]** and ***trans*[3]** show the similar redox peaks.

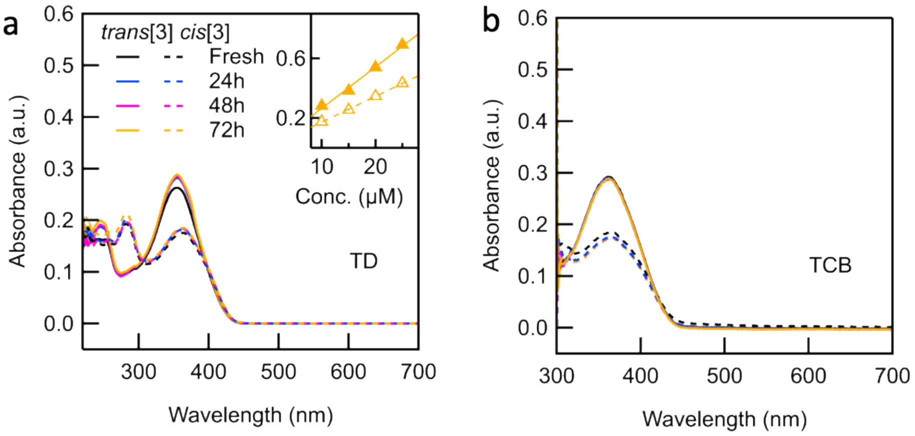

Supplementary Figure 2. Optical absorption characterizations. (a) UV-vis absorption spectra of ***cis*[3]** and ***trans*[3]** measured in tetradecane. The solutions were kept in the dark at room temperature for 72 hours and the UV-vis spectra were recorded at different times period. In 72 hours, there is no obvious shift of the absorption peaks of ***cis*[3]** and ***trans*[3]**. Inset: Absorbance at 350 nm for a solution of ***cis*[3]** and ***trans*[3]** in TD at different concentrations. The extinction coefficient of ***cis*[3]** and ***trans*[3]** is determined be 17300 and 27510 respectively by fitting of the absorbance versus concentration data. (b) UV-vis absorption spectra of ***cis*[3]** and ***trans*[3]** in TCB with a concentration of 10 μM.

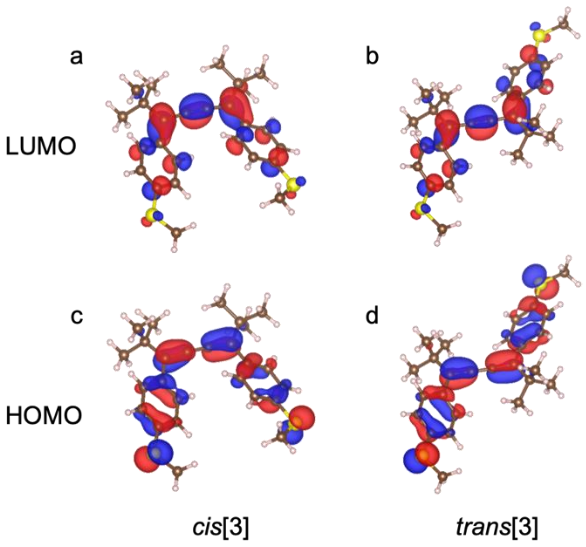

Supplementary Figure 3. Frontier orbital energy level calculations. (a) LUMO and (c) HOMO orbitals of ***cis*[3].** (b) LUMO and (d) HOMO orbitals of ***trans*[3].** The two isomers show the same LUMO and HOMO energy levels of -1.9 and -5.3 eV.

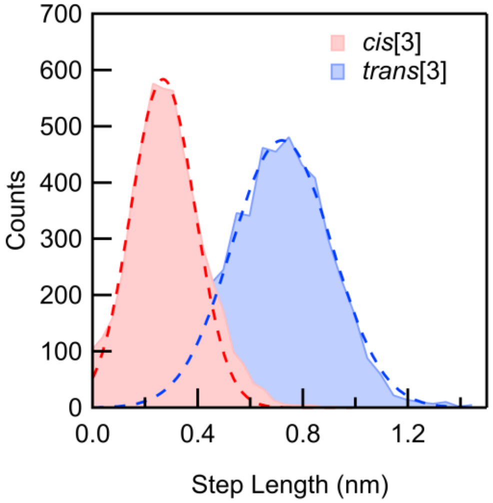

Supplementary Figure 4. Molecular conductance plateau length analysis. Step length histograms for ***cis*[3]** and ***trans*[3]** conductance data measured in TD. Step lengths are determined by counting the number of data points within a conductance range of 10^{-3} to $10^{-4.9}$ G_0 in 5000 conductance traces. ***cis*[3]** and ***trans*[3]** show distinct conductance plateau length distributions with a mean of 0.26 nm and 0.75 nm (determined by Gaussian fit). The fraction of traces that have a plateau longer than 0.1 nm is 94% for ***cis*[3]** and ~98% for ***trans*[3]**.

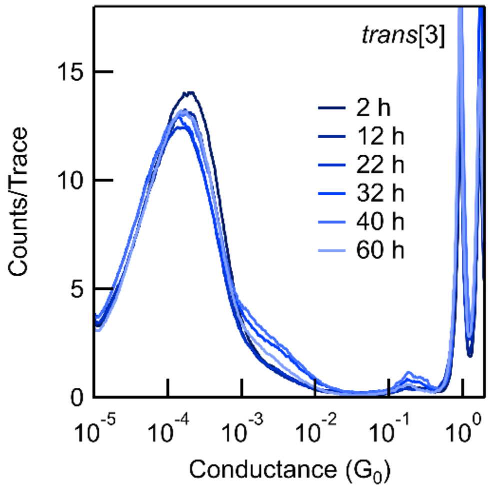

Supplementary Figure 5. Conductance histogram analysis of ***trans*[3]**. Logarithmically-binned 1D histograms as a function of time for ***trans*[3]** measurement in tetradecane at a bias of 0.1 V. Each histogram is compiled from 10000 conductance traces measured within 2 hours at different times over the 60 hour experiment. These results show that the conductance features of ***trans*[3]** do not change during the measurements.

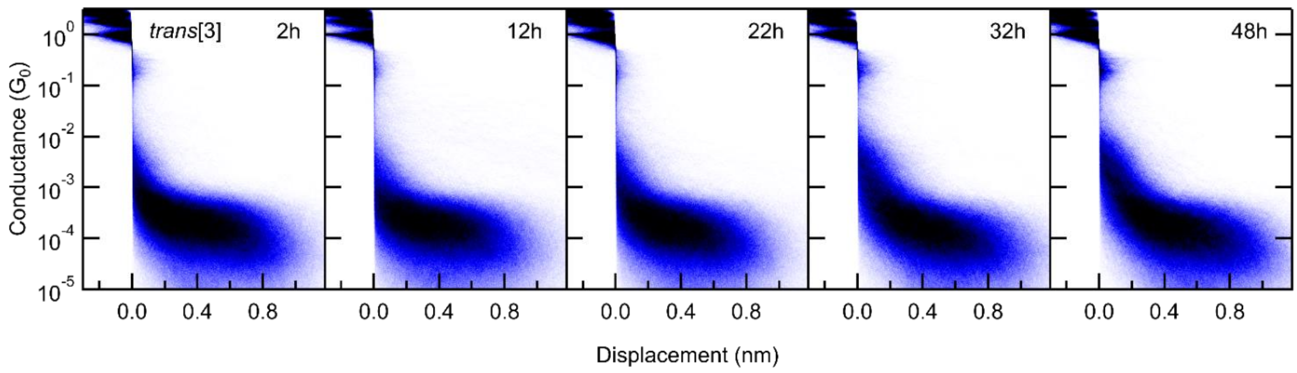

Supplementary Figure 6. Conductance histogram analysis of ***trans*[3]**. 2D conductance-displacement histograms as a function of time for ***trans*[3]** measurement in tetradecane at a bias of 0.1 V. Each histogram is compiled from the same data shown in **Supplementary Figure 5.** Conductance histogram analysis of ***trans*[3]**. These results show that the conductance features of ***trans*[3]** do not change during the measurements.

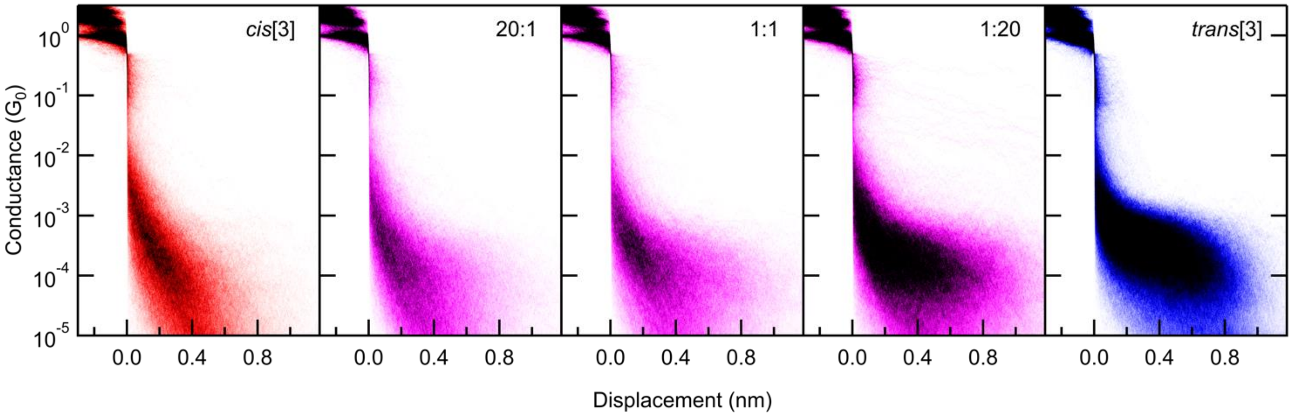

Supplementary Figure 7. Conductance histogram analysis of mixture of ***cis*****[3]** and ***trans*****[3]**. 2D conductance-displacement histograms obtained under a tip bias of 0.1 V for mixture of ***cis*****[3]** and ***trans*****[3]** in tetradecane with different ratios. The histogram is generated by compiling 5000 traces collected in ~ 1 hour for each measurement.

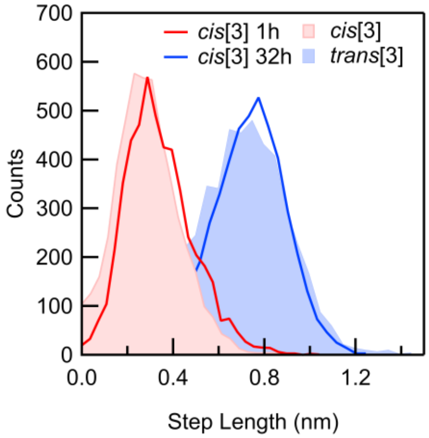

Supplementary Figure 8. Molecular conductance plateau length analysis. 1D step length distribution histograms of ***cis*****[3]** determined from 5000 conductance traces collected in the 1st hour (red) and 32nd hour (blue) of the measurements. Also shown are the ***cis*****[3]** (filled pink) and ***trans*****[3]** (filled blue) histograms from Figure S4. It is clear that the step length distribution determined from the ***cis*****[3]** measurement after 32 hours coincides very well with that from a pure ***trans*****[3]** measurement. We therefore estimate a >90% yield of ***trans*****[3]** product in the solution after 32 hr.

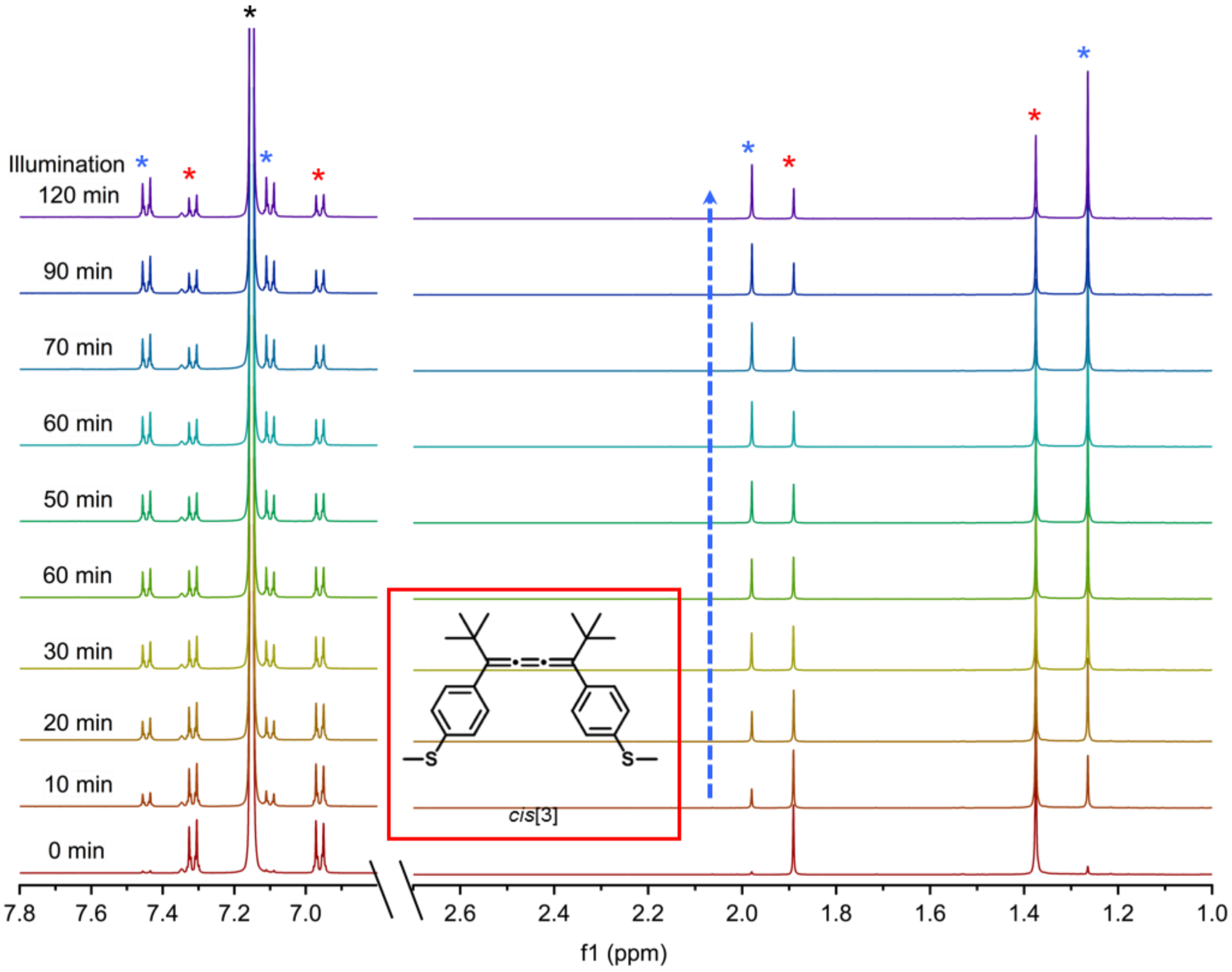

Supplementary Figure 9. Time dependent 1H NMR (500 MHz) spectra analysis of ***cis*****[3].** Time dependent ^{1}H NMR (500 MHz) spectra of ***cis*****[3]** showing regions of the aromatic (7.5-6.9 ppm), the SMe (2.0-1.8 ppm), and the *t*Bu (1.4-1.2 ppm) groups (measured in benzene-d_6) upon illumination (with white light). The low field (7.5-6.9 ppm) region is expanded for clarity. The signature peaks of ***cis*****[3]** are denoted by the red stars. The peak denoted by the black star is assigned to the benzene-d_6 solvent. After illuminating for 10 min, the signature peaks of ***trans*****[3]** (denoted by the blue stars) occur due to photoisomerization. After 60 min of illumination, spectra show a pair of well-resolved signature peaks of ***cis*****[3]** and ***trans*****[3]** which do not change upon further illumination. By fitting the peak areas, we can determine the photostationary equilibrium ***cis*****[3]**:***trans*****[3]** ratio to be ~40:60 in the solution.

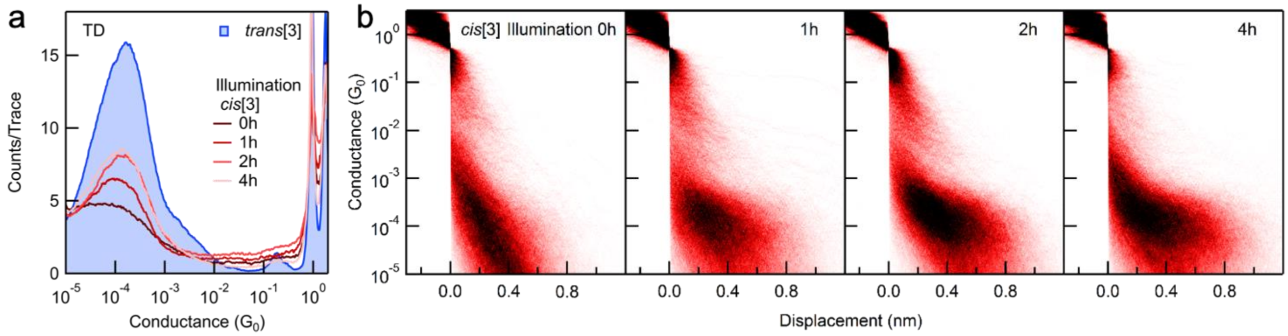

Supplementary Figure 10. Conductance histogram analysis of ***cis*****[3].** (a) Logarithmically-binned 1D histograms of ***cis*****[3]** in TD as a function of white light illumination time. The applied bias is 0.1 V. Each histogram is compiled from 5000 conductance traces generated within 1 hour at different time periods over the measurement timescale. The histogram of ***trans*****[3]** (filled blue) is shown for comparison. (b) 2D conductance-displacement histograms compiled from the same data. We see photoisomerization which yields a mixture of ***cis*****[3]** and ***trans*****[3]** in the solution. These are in good agreement with the ^{1}H NMR characterizations shown in **Supplementary Figure 9**.

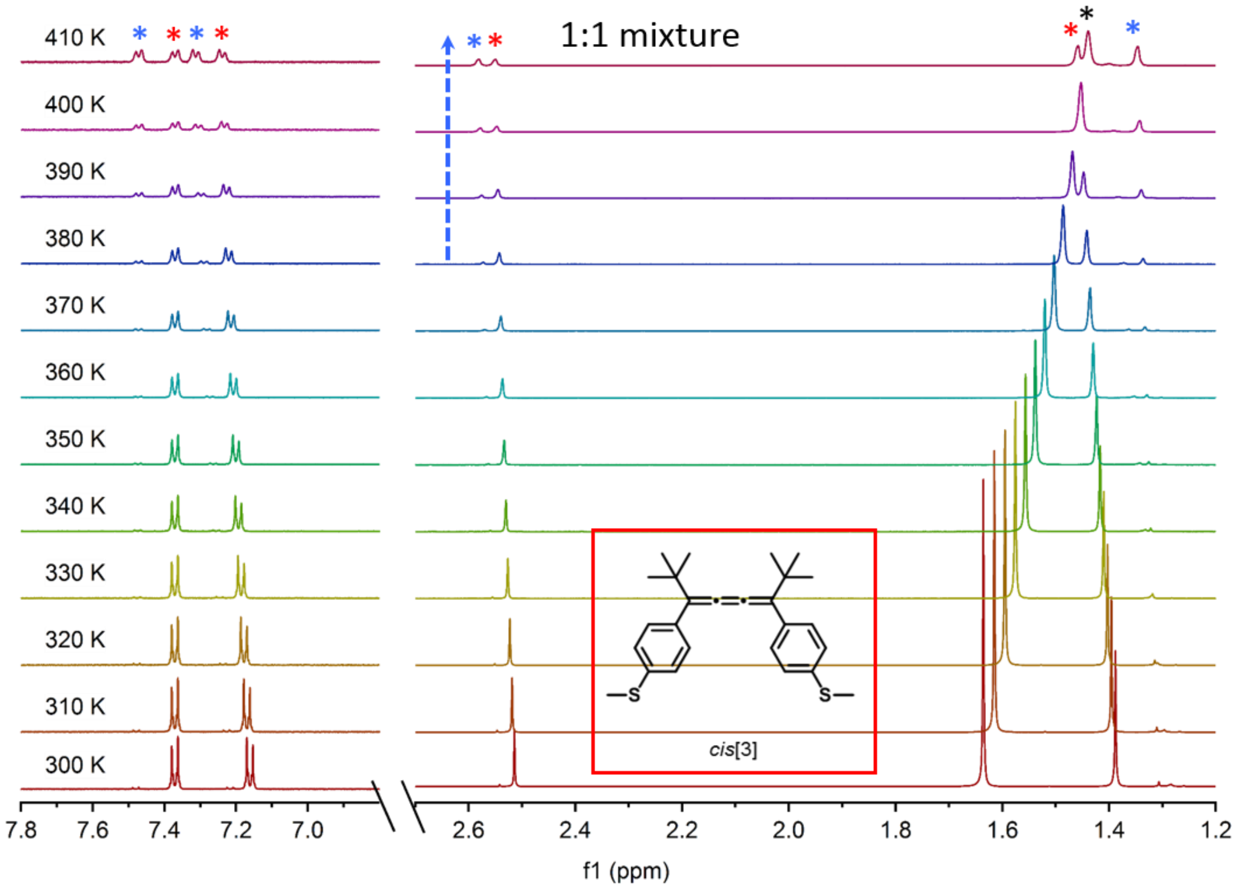

Supplementary Figure 11. Varying temperature ^{1}H NMR (500 MHz) spectra analysis of ***cis*[3].** Varying temperature ^{1}H NMR (500 MHz) spectra of ***cis*[3]** showing regions of the aromatic (7.5-7.1 ppm), the SMe (2.6-2.5 ppm), and the *t*Bu (1.5-1.2 ppm) groups (measured in $C_2D_2Cl_4$). The low field (7.5-7.1 ppm) region is expanded for clarity. The signature peaks of ***cis*[3]** are denoted by the red stars. The peak denoted by the black star is assigned to a water impurity in the solution. When the temperature is increased to 380 K, the signature peaks of ***trans*[3]** (denoted by the blue stars) are seen due to a thermal isomerization. At 410 K, the spectra show a pair of well-resolved peaks of ***cis*[3]** and ***trans*[3]** with the same peak areas, indicating a near equimolar distribution of the two isomers in the solution. Note that by simply using temperature or light to catalyze the reaction, one can only obtain a 50:50 ratio of *cis:trans*.

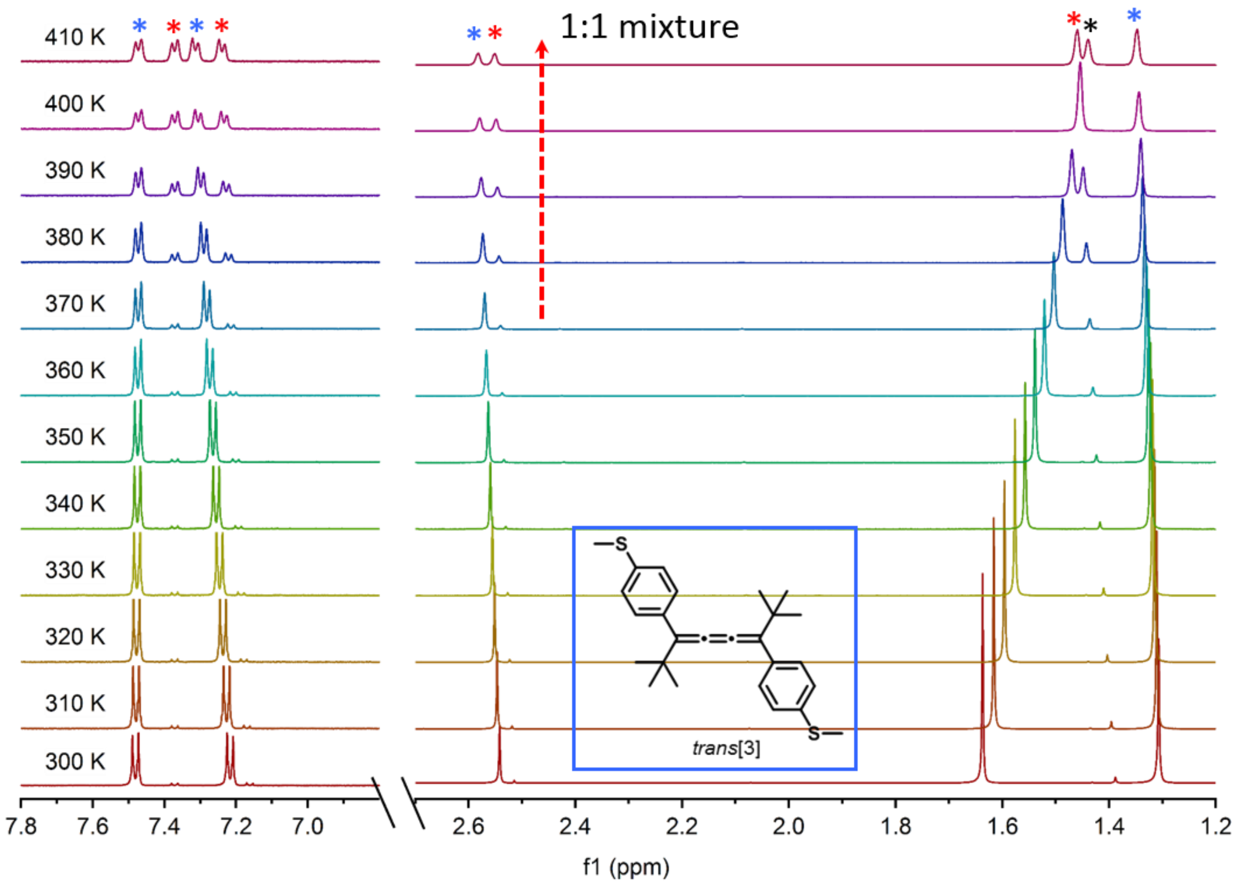

Supplementary Figure 12. Varying temperature ^{1}H NMR (500 MHz) spectra analysis of ***trans*****[3].** Varying temperature ^{1}H NMR (500 MHz) spectra of ***trans*****[3]** showing regions of the aromatic (7.5-7.1 ppm), the SMe (2.6-2.5 ppm), and the *t*Bu (1.5-1.2 ppm) groups (measured in $C_2D_2Cl_4$). The low field (7.5-7.1 ppm) region is expanded for clarity. The signature peaks of ***trans*****[3]** are denoted by the blue star. The peak denoted by the black star is assigned to a water impurity in the solution. When the temperature is increased to 370 K, the signature peaks of ***cis*****[3]** (denoted by the red star) are seen due to a thermal isomerization. At 410 K, the spectra show a pair of well-resolved signature peaks of ***cis*****[3]** and ***trans*****[3]** with the same peak areas, indicating a near equimolar distribution of the two isomers in the solution. This thermodynamic equilibrium indicates that the two isomers have approximately the same free energy. These NMR spectra still show two clear peaks up to 410 K, indicating that the conversion barrier is large (likely greater than 1 eV). These measurements indicate that the ***cis*****[3]** and ***trans*****[3]** are stable and do not interconvert within experimental time-scales at room temperature.

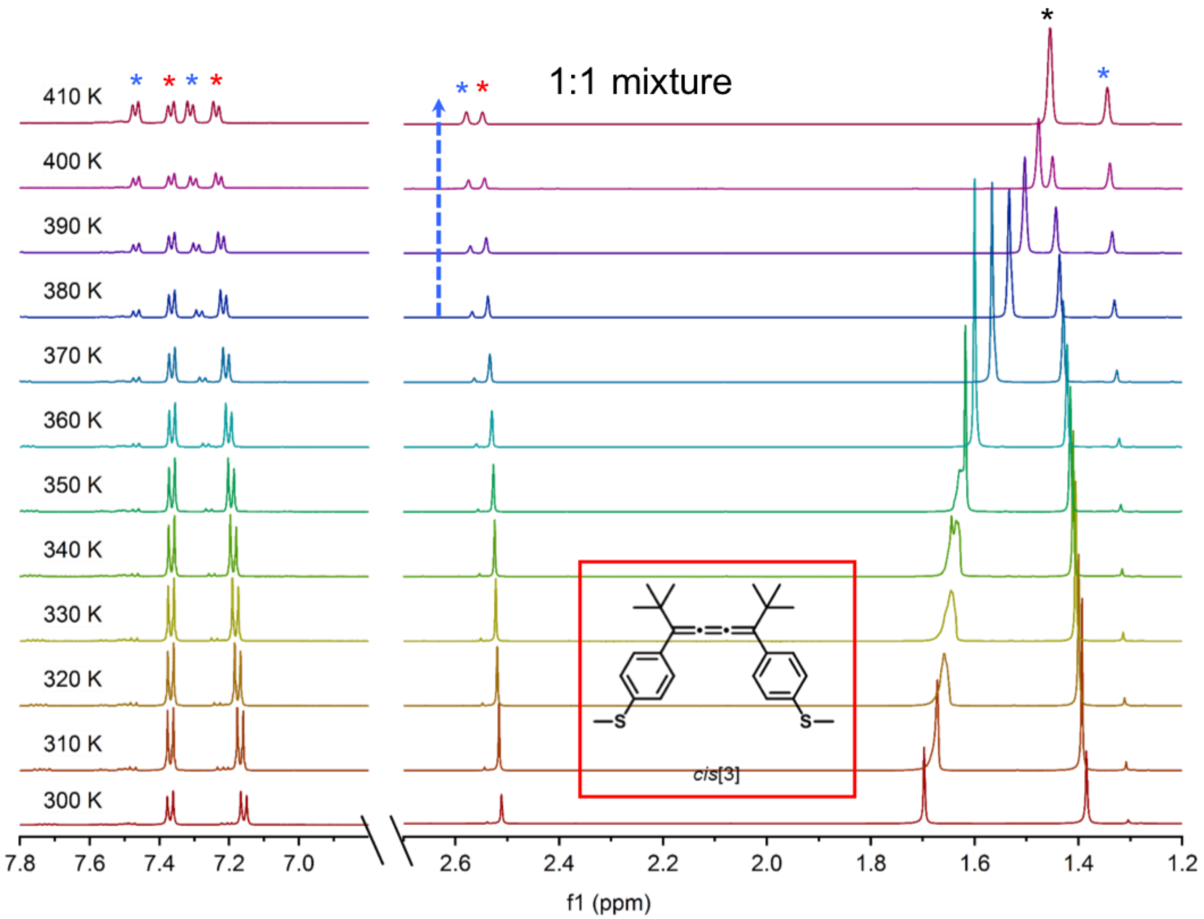

Supplementary Figure 13. Varying temperature ^{1}H NMR (500 MHz) spectra analysis of ***cis*[3].** Varying temperature ^{1}H NMR (500 MHz) spectra of ***cis*[3]** (3.0 mg) with 10 μL gold nanoparticles (2 nm, 0.01% Au aqueous solution) showing regions of the aromatic (7.5-7.1 ppm), the SMe (2.6-2.5 ppm), and the *t*Bu (1.5-1.2 ppm) groups (measured in $C_2D_2Cl_4$). The low field (7.5-7.1 ppm) region is expanded for clarity. The signature peaks of ***cis*[3]** are denoted by the red stars. The peak denoted by the black star is assigned to a water impurity in the solution. When the temperature is increased to 380 K, the signature peaks of ***trans*[3]** (denoted by the blue star) are seen due to a thermal isomerization. At 410 K, the spectra show a pair of well-resolved signature peaks of ***cis*[3]** and ***trans*[3]** with the same peak areas, indicating a near equimolar distribution of the two isomers in the solution.

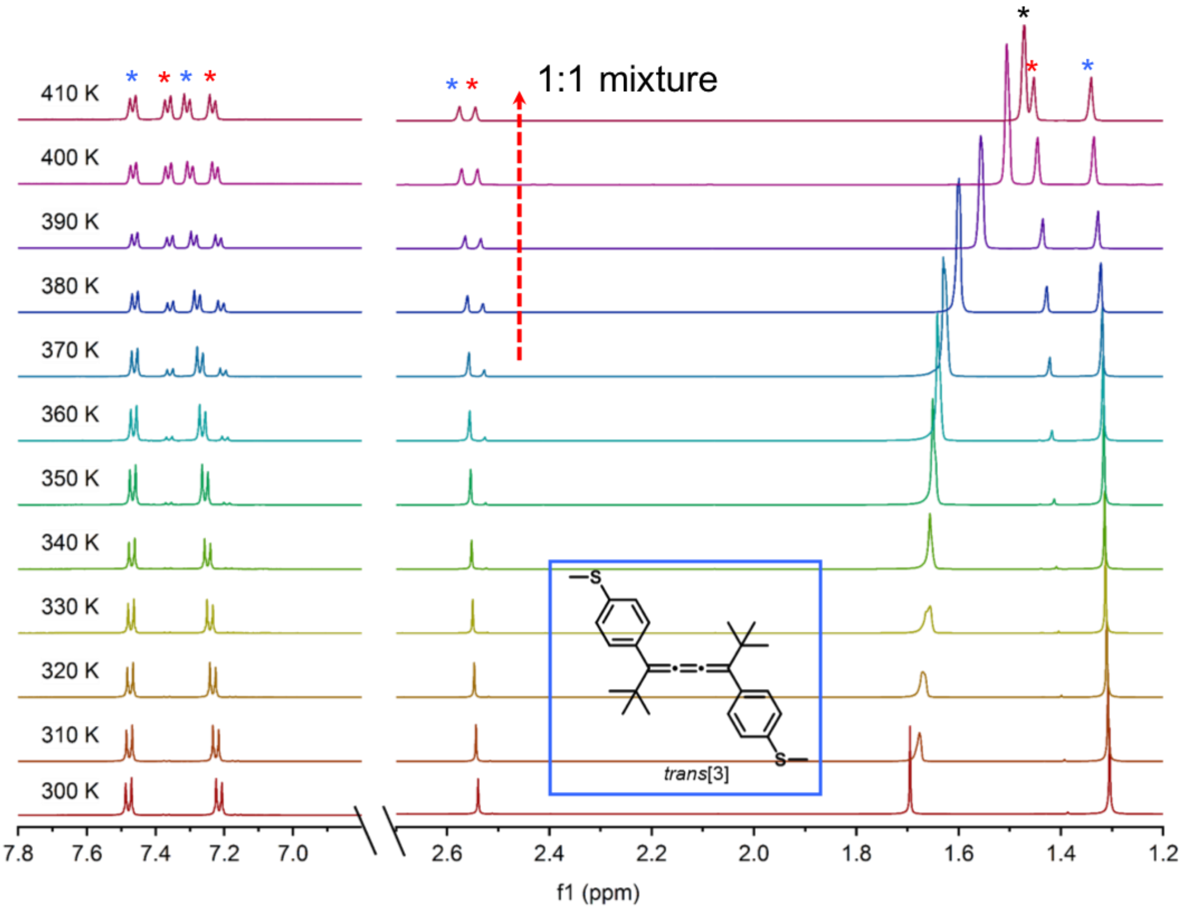

Supplementary Figure 14. Varying temperature ^{1}H NMR (500 MHz) spectra analysis of ***trans*[3].** Varying temperature ^{1}H NMR (500 MHz) spectra of ***trans*[3]** (3.0 mg) with 10 µL of gold nanoparticles (2 nm, 0.01% Au aqueous solution) showing regions of the aromatic (7.5-7.1 ppm), the SMe (2.6-2.5 ppm), and the *t*Bu (1.5-1.2 ppm) groups (measured in $C_2D_2Cl_4$). The low field (7.5-7.1 ppm) region is expanded for clarity. The signature peaks of ***trans*[3]** are denoted by a blue star. The peak denoted by a black star is assigned to a water impurity in the solution. When the temperature is increased to 370 K, the signature peaks of ***cis*[3]** (denoted by the red stars) are seen due to a thermal isomerization. At 410 K, the spectra show a pair of well-resolved signature peaks of ***cis*[3]** and ***trans*[3]** with the same peak areas, indicating a near equimolar distribution of the two isomers in the solution. These behaviors are consistent with those shown in **Supplementary Figures 11-12** indicating that adding gold nanoparticles does not affect the temperature at which the isomerization occurs. This proves that the charge transfer between gold and the linkers of cumulene molecules is not the dominated factor for the *cis*-to-*trans* isomerization observed in the STM-BJ measurements.

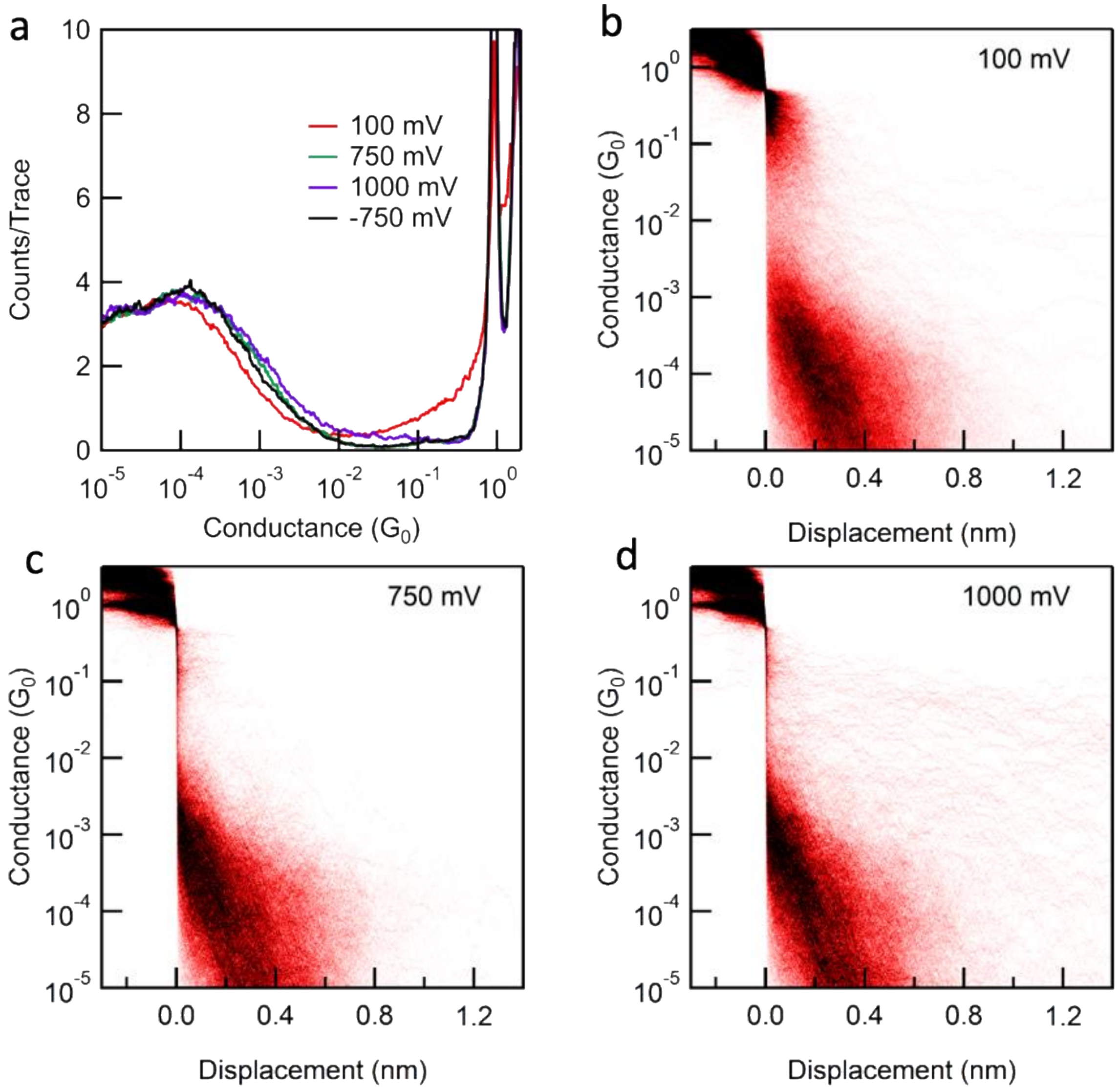

Supplementary Figure 15. Conductance histogram analysis of ***cis*[3].** (a) Logarithmically-binned 1D histograms for ***cis*[3]** in TD obtained at a bias of 0.1 V (red), 0.75 V (green), 1V (purple) and -0.75 V (black). Each histogram is compiled from 5000 consecutively measured traces without any data selection. (b-d) 2D conductance-displacement histograms compiled from the same data. The conductance does not change with bias voltage, indicating an off-resonant transport mechanism in this voltage range.

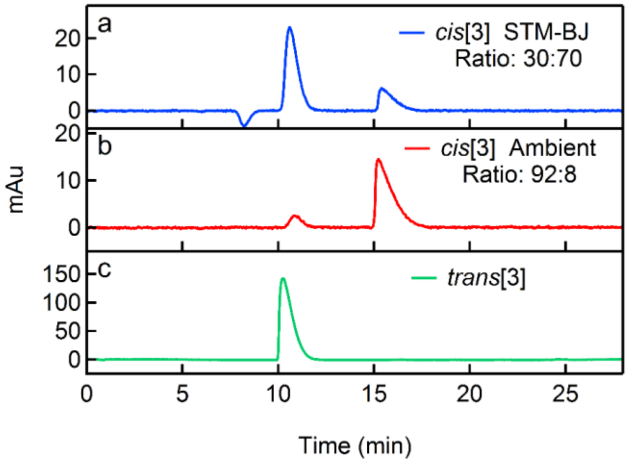

Supplementary Figure 16. HPLC analysis. HPLC data collected for (a) ***cis*[3]** solution in TD taken from the STM-BJ setup after running the measurement for ~ 240 hrs at a 0.3 V bias, (b) same solution kept outside the setup and (c) a solution of the pure ***trans*[3]** compound. The *trans* isomer is visible at around 10 minutes while the *cis* is seen at around 16 minutes. To determine the *cis* to *trans* ratios in the measured solution, we first obtain the extinction coefficients for *cis* and *trans* from the UV-vis data (inset of **Supplementary Figure 2**) We obtain 17300 for *cis* and 27510 for *trans*. The *cis:trans* ratio is determined from the the HPLC spectra as:

$$\text{STM-BJ:}\frac{A_{cis}/17300}{A_{trans}/27510} \approx 30:70$$

$$\text{Ambient: }\frac{A_{cis}/17300}{A_{trans}/27510} \approx 92:8$$

where A_{cis} and A_{trans} are the integrated areas of *cis* and *trans* signature peaks in the HPLC data.

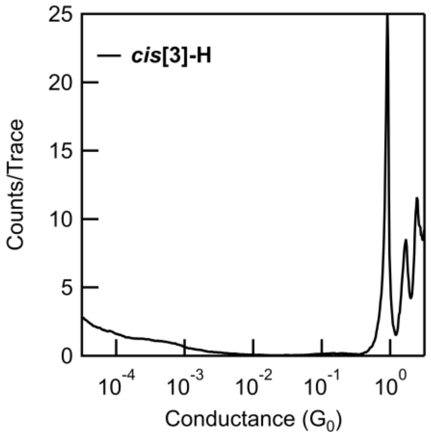

Supplementary Figure 17. Conductance histogram analysis of ***cis*[3]-H.** Histogram of 5000 traces measured in a ***cis*[3]-H** solution in TD at 0.5 V bias. No molecular conductance peaks are visible.

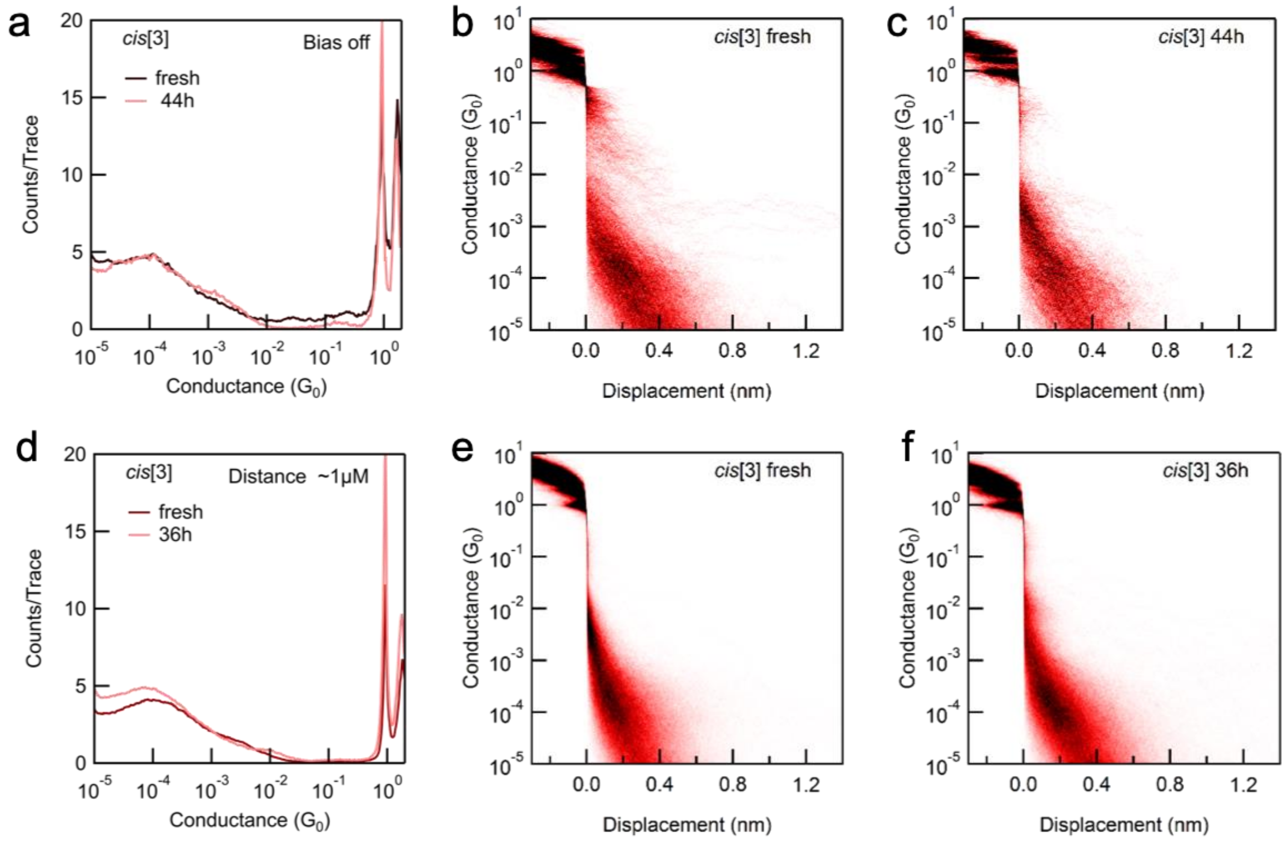

Supplementary Figure 18. Conductance histogram analysis of ***cis*[3].** (a) Histogram of 1000 traces measured in a fresh ***cis*[3]** solution in TD (brown). Following this measurement, the bias was turned off for 2 hr periods and 1000 traces were measured again. This cycle was repeated and the histogram from the last measurement after 44 hrs is shown in pink. The corresponding 2D histograms are shown in panel (b) and (c). (d) Histogram of 1000 traces measured in a fresh ***cis*[3]** solution in TD (brown). Following this measurement, the tip was retracted by ~1 μm for 2 hr periods and 1000 traces were measured again. This cycle was repeated and the histogram after the last cycle in the same solution is shown in pink. The corresponding 2D histograms are shown in panel (e) and (f).

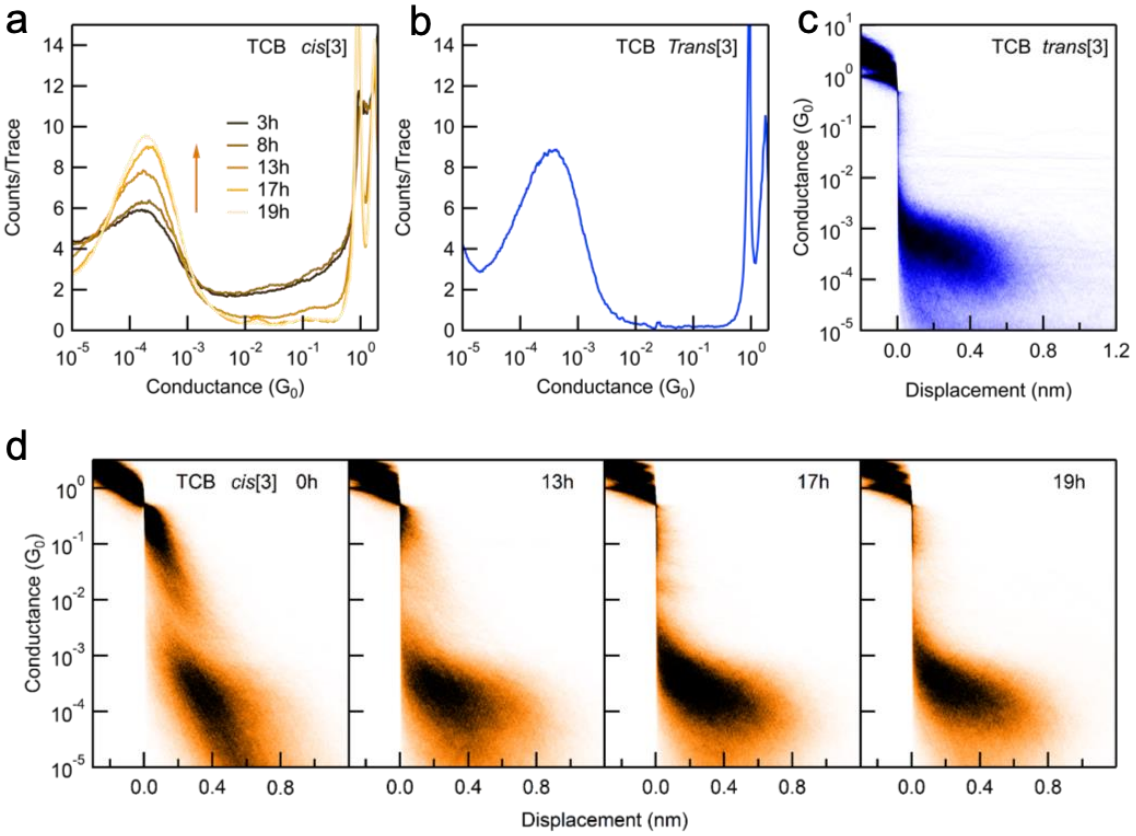

Supplementary Figure 19. Conductance histogram analysis of ***cis*[3].** (a) Logarithmically-binned 1D histograms as a function of time for ***cis*[3]** in TCB obtained at a tip bias of 0.1 V. Each histogram is compiled from 10000 conductance traces measured in 2 hours at different times as indicated. (b) Logarithmically-binned 1D histograms for ***trans*[3]** in TCB compiled from 5000 traces generated within 1 hours at a tip bias of 0.1 V. (c) and (d) 2D conductance-displacement histograms compiled from the data shown in (a) and (b). A similar *cis*-to-*trans* isomerization is clearly seen as the conductance feature evolves with time.

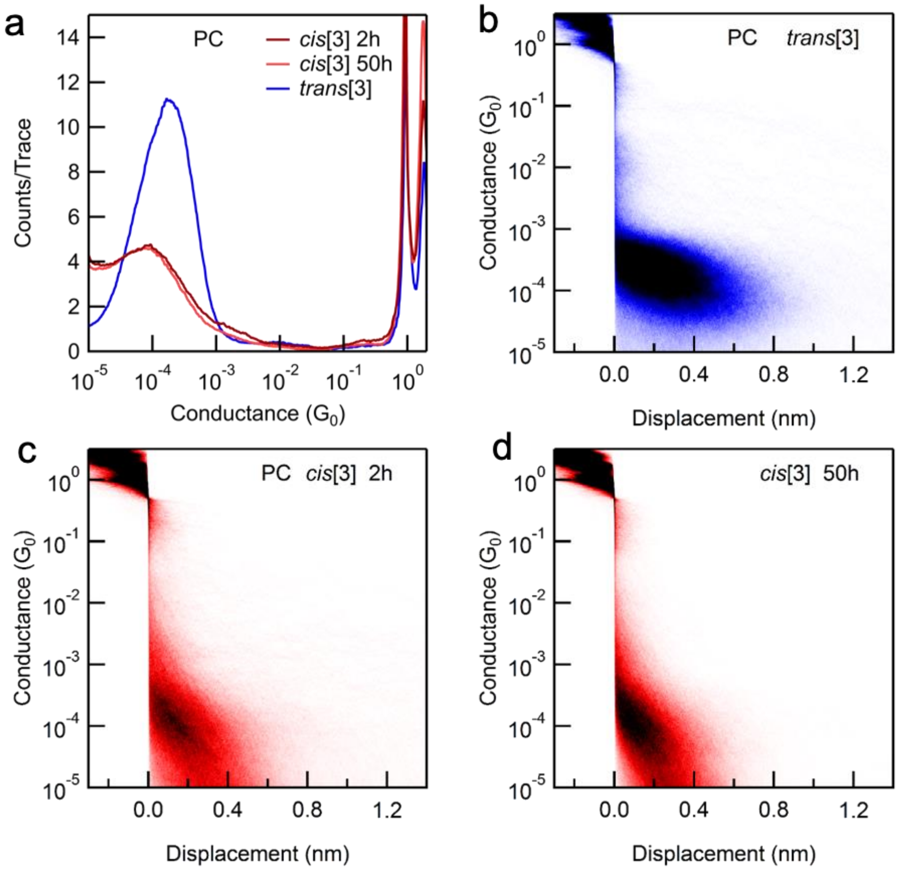

Supplementary Figure 20. Conductance histogram analysis of ***cis*[3].** (a) Logarithmically-binned 1D histograms for ***cis*[3]** and ***trans*[3]** in PC with supporting electrolyte obtained at a tip bias of 0.1 V. The histograms of ***cis*[3]** are compiled from 10000 conductance traces generated within 2 hours at different time periods over the measurement timescale (red and pink). The histogram of ***trans*[3]** is compiled from 10000 conductance traces generated within 2 hours of a fresh solution (blue). (b) 2D conductance-displacement histograms compiled from the same data (b) for ***trans*[3]** and (c, d) ***cis*[3]**. We note that the conductance features of ***cis*[3]** are not changing with time, indicating that there is no obvious *cis*-to-*trans* isomerization taking place over time in polar solvent.

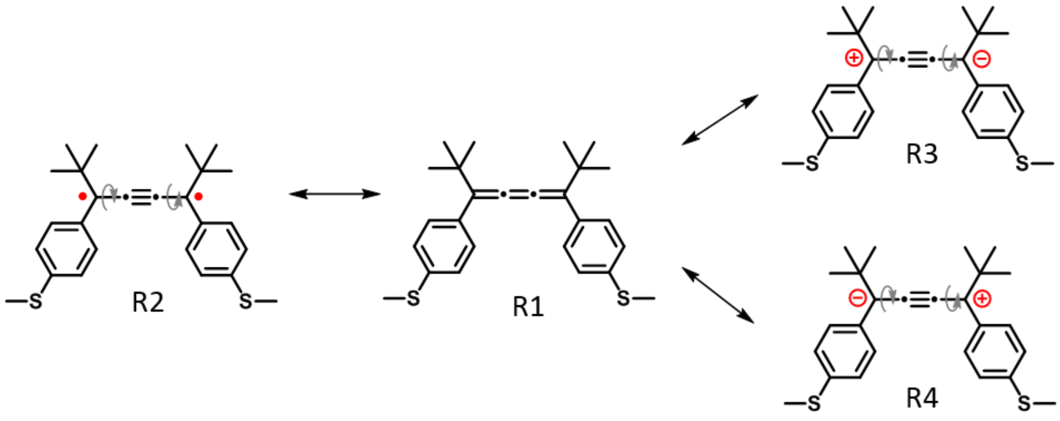

Supplementary Figure 21. Electronic structure analysis of ***cis*****[3].** Resonance structures of ***cis*****[3]**. R1 is the standard canonical resonance form. A simple recoupling of electronic spins gives the 1,4-diradical resonance form (R2), while electrostatic polarization gives the zwitterionic resonance forms, R3 and R4. Although R1 is used as shorthand, a full understanding of cumulenes requires the inclusion of the other canonical forms. The important feature common to the structures of R2, R3 and R4 is the (relatively) free rotation around the terminal C-C bonds, since the terminal π-bonds are absent in these forms. Thus, any physical effect that would promote these "alkyne" resonance forms would reduce the barrier to rotational isomerization.

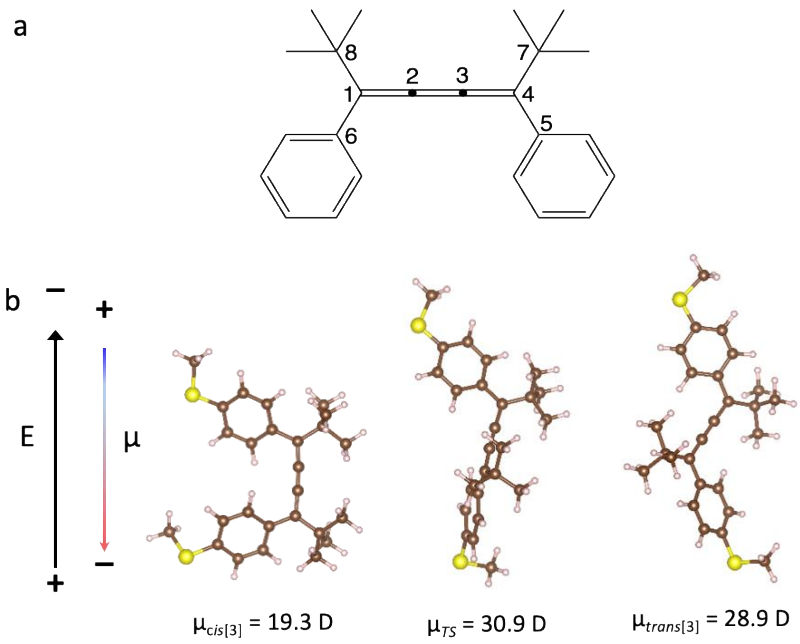

Supplementary Figure 22. Density functional theory calculations. (a) Structure of ***cis*[3]** indicating carbon atom labels. (b) Schematic illustration of the orientation of molecules under a field of 5 V/nm in the ground and transition state. The field induced dipole moments are indicated.

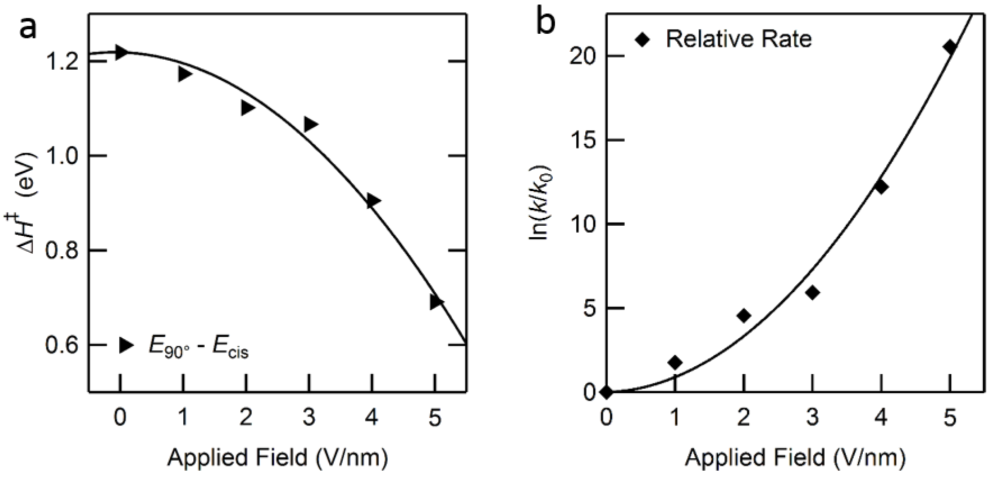

Supplementary Figure 23. Density functional theory calculations. (a) the calculated activation energy and (b) natural logarithm of the relative rate at room temperature as a function of the applied electric field ($e^{-(\Delta H^{\ddagger}-\Delta H^{\ddagger}_{Field})/kT}$).

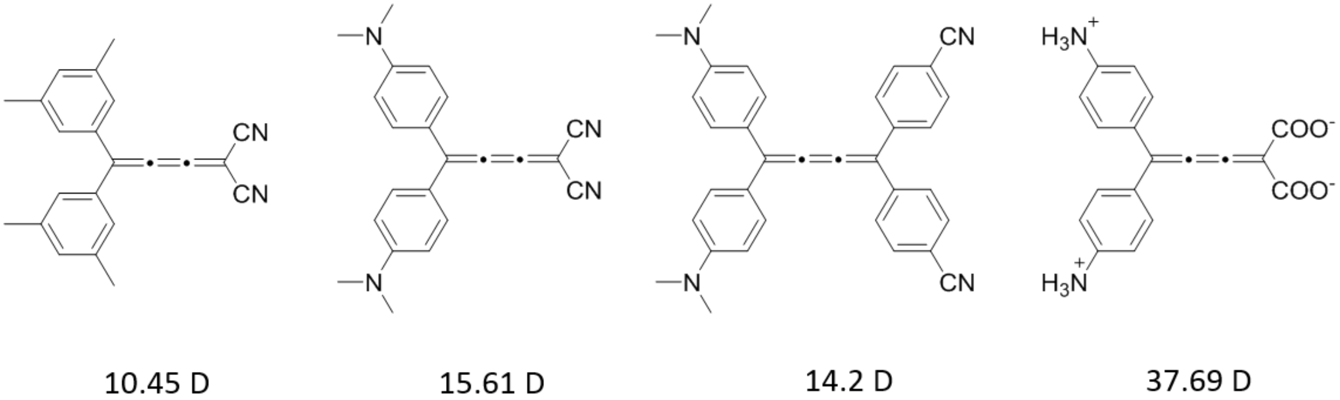

Supplementary Figure 24. Density functional theory calculations. Molecular structure of [3]cumulene compounds with different donor and acceptor substitutions and their intrinsic dipole moments.

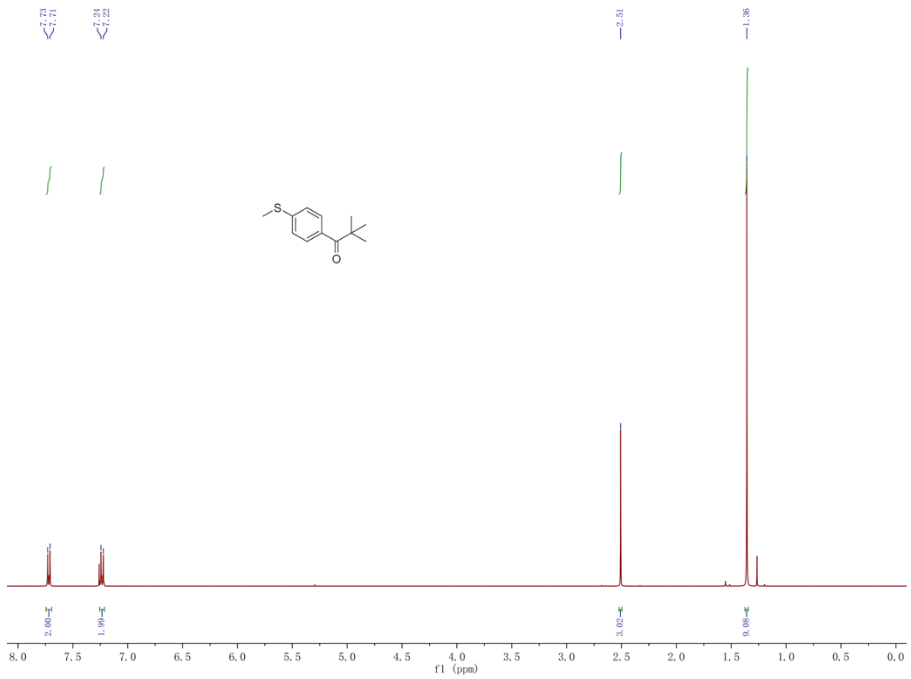

Supplementary Figure 25. ^{1}H NMR spectrum of **1**.

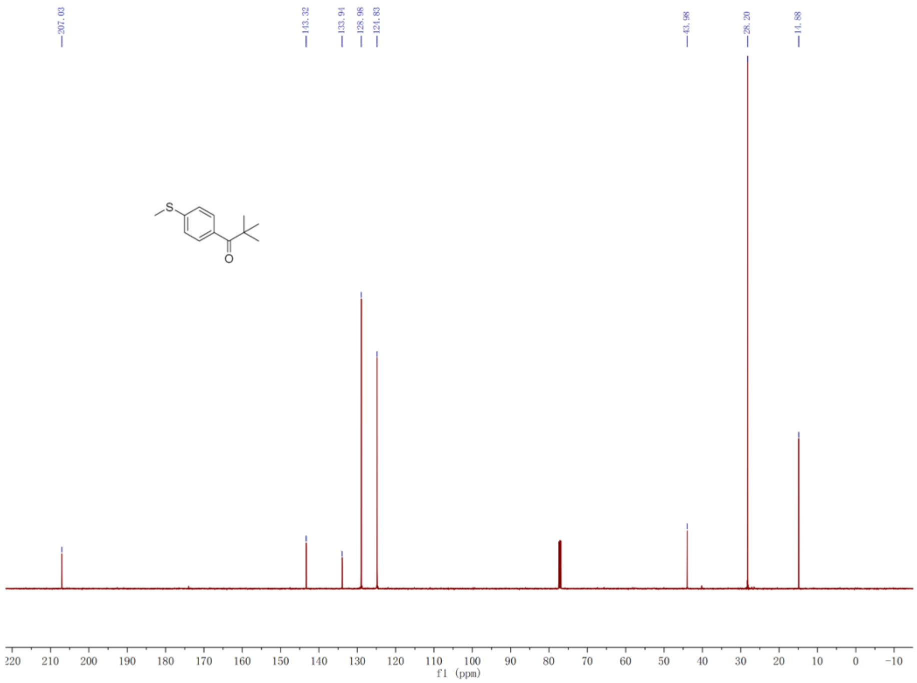

Supplementary Figure 26. ^{13}C NMR spectrum of **1**.

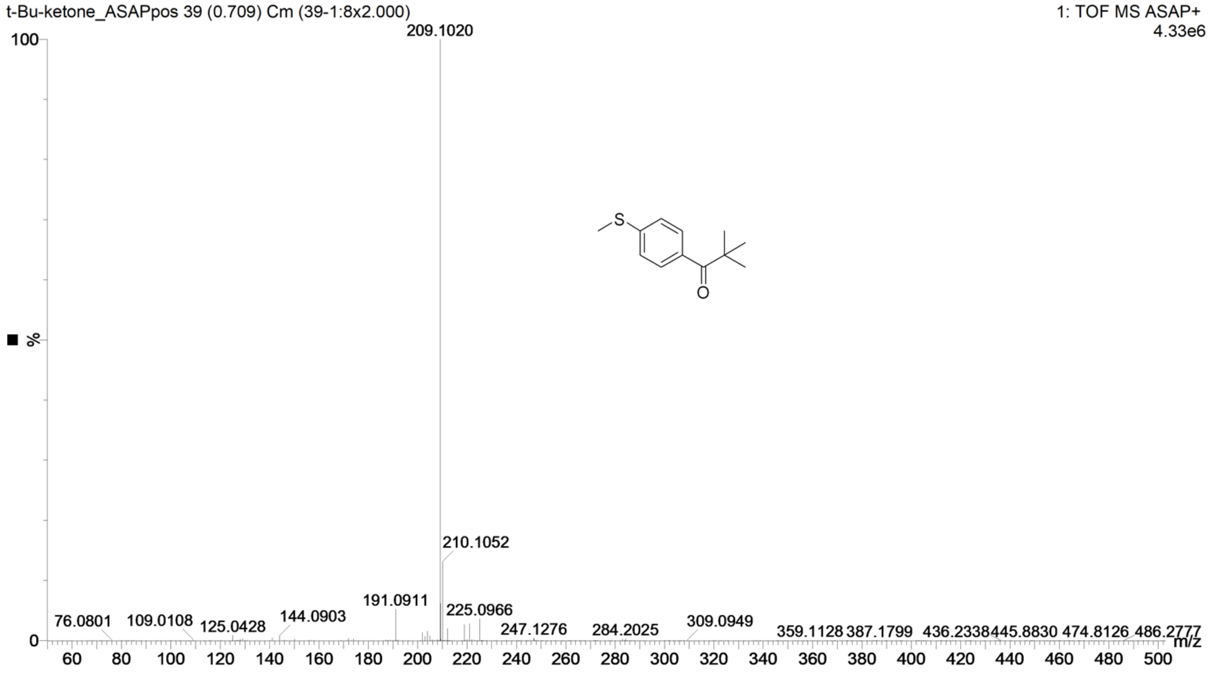

Supplementary Figure 27. HRMS of **1.**

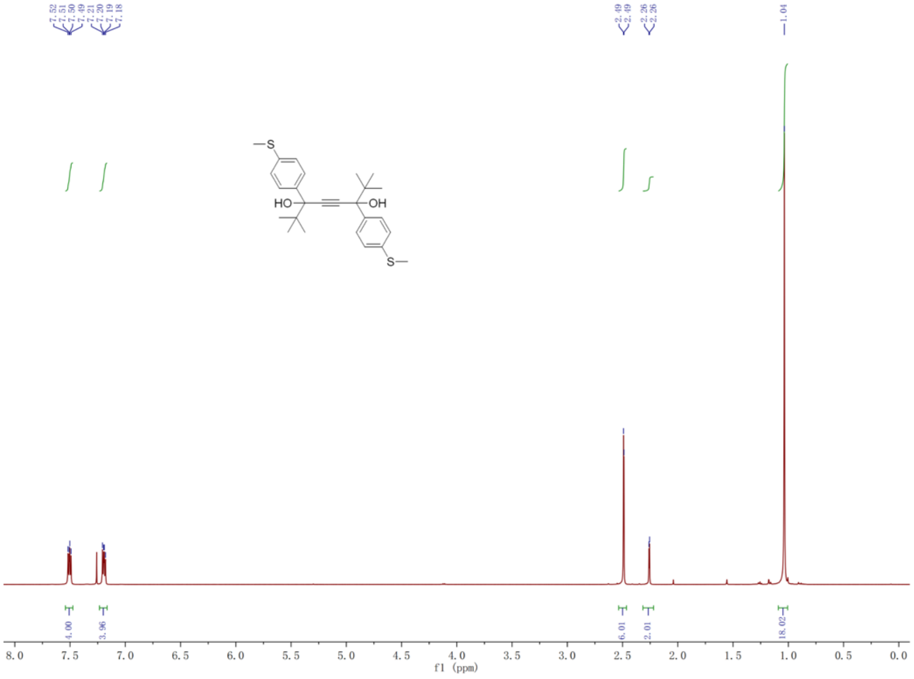

Supplementary Figure 28. ^{1}H NMR spectrum of **2**.

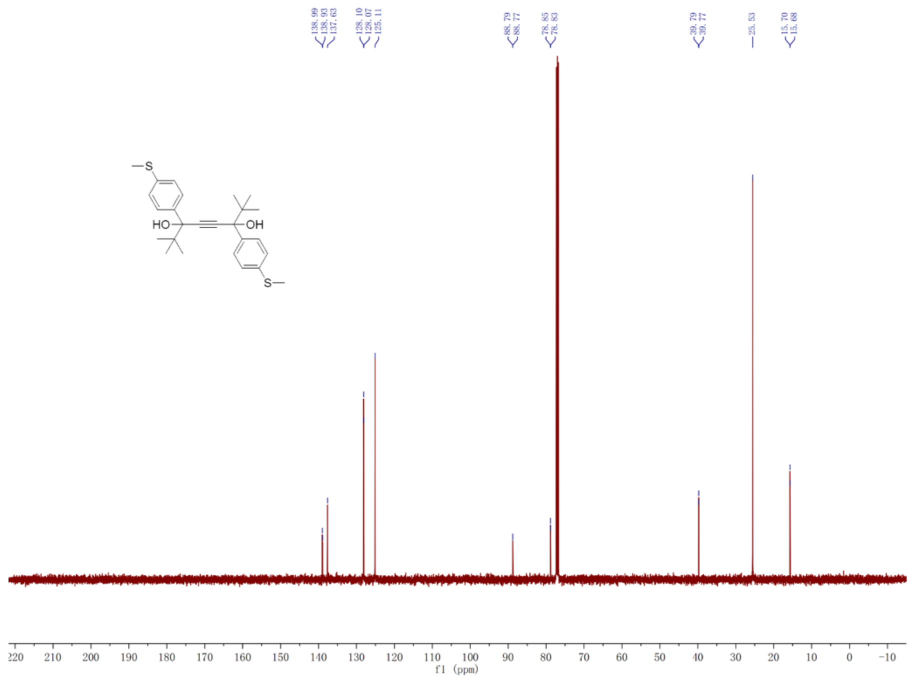

Supplementary Figure 29. ^{13}C NMR spectrum of **2**.

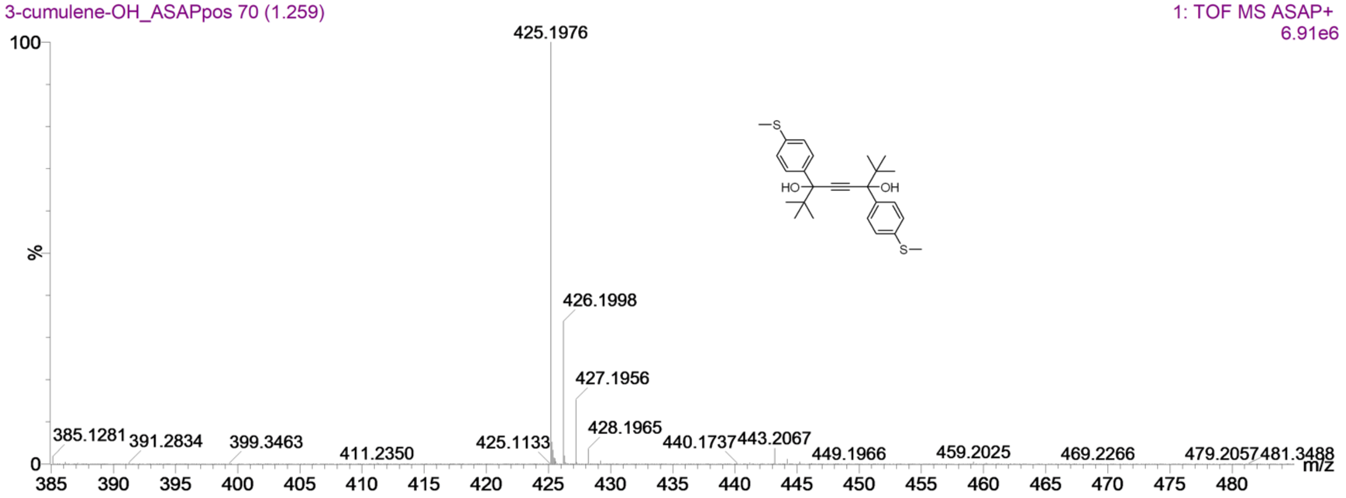

Supplementary Figure 30. HRMS of **2**.

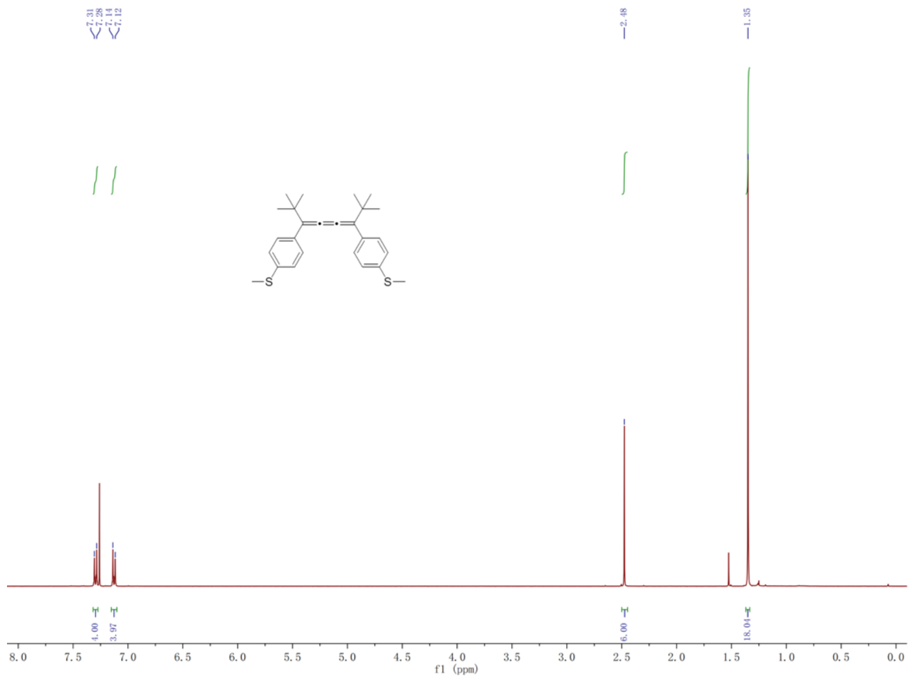

Supplementary Figure 31. ^{1}H NMR spectrum of ***cis*[3]**.

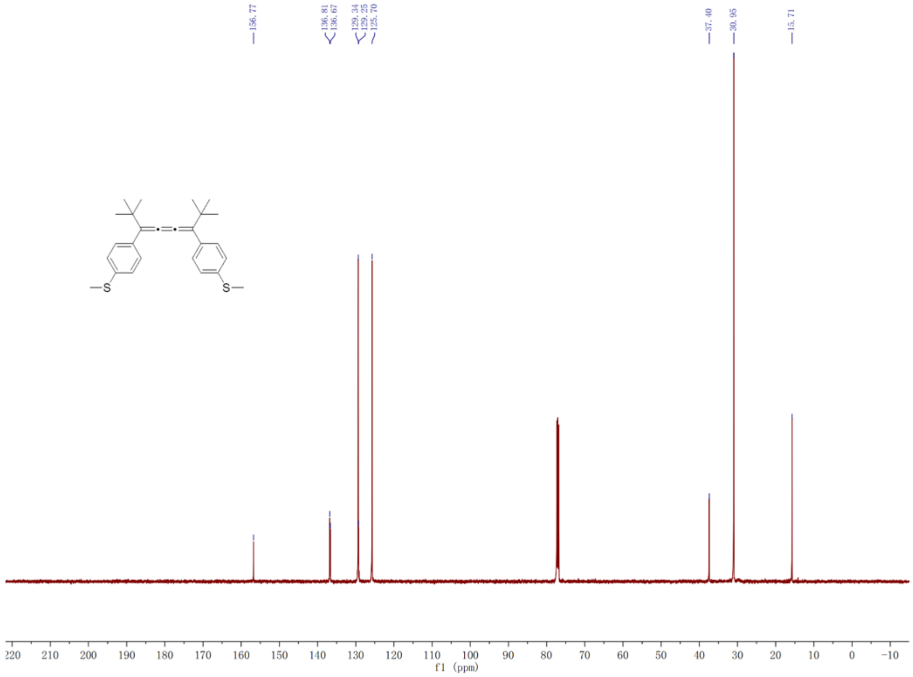

Supplementary Figure 32. ^{13}C NMR spectrum of ***cis*[3]**.

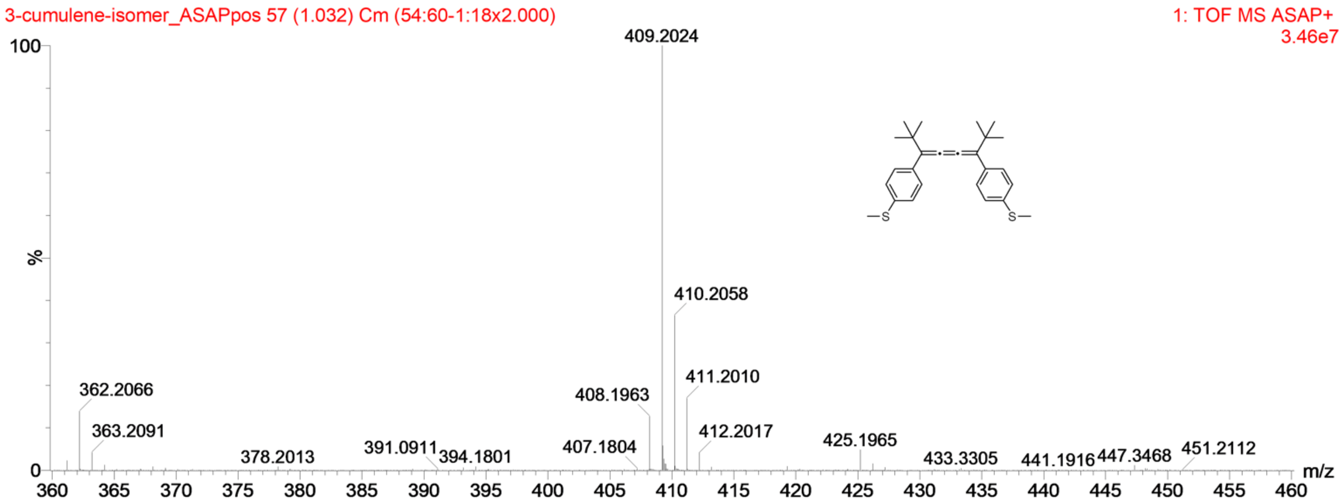

Supplementary Figure 33. HRMS of ***cis*[3]**.

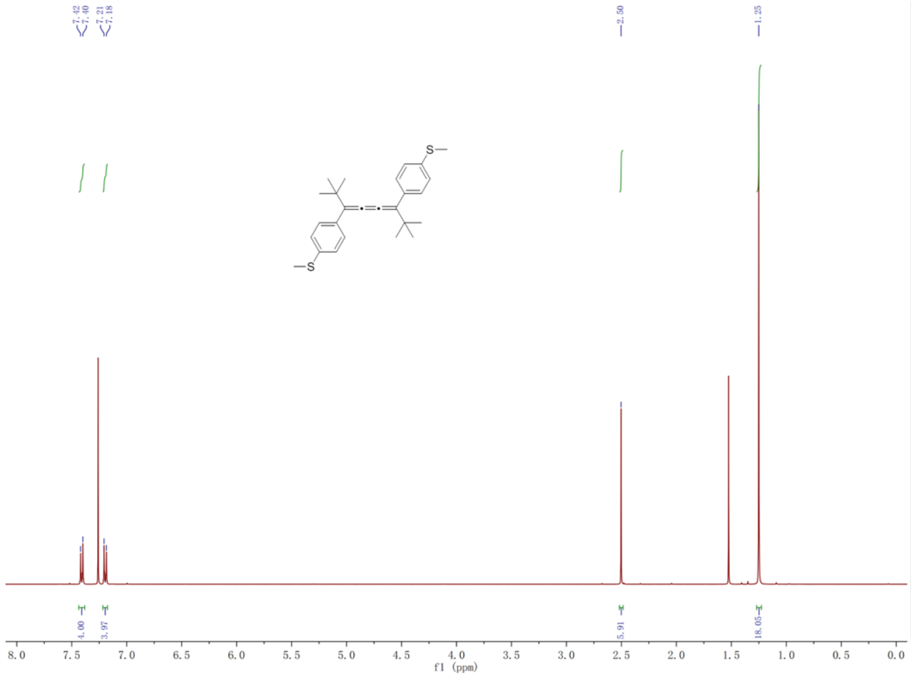

Supplementary Figure 34. ^{1}H NMR spectrum of ***trans*[3]**.

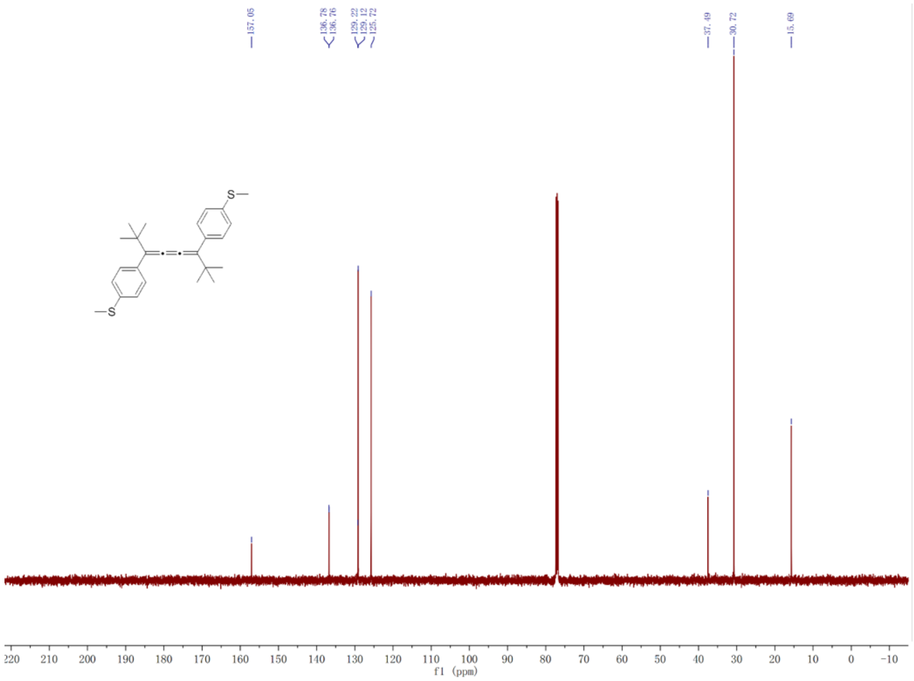

Supplementary Figure 35. ^{13}C NMR spectrum of ***trans*[3]**.

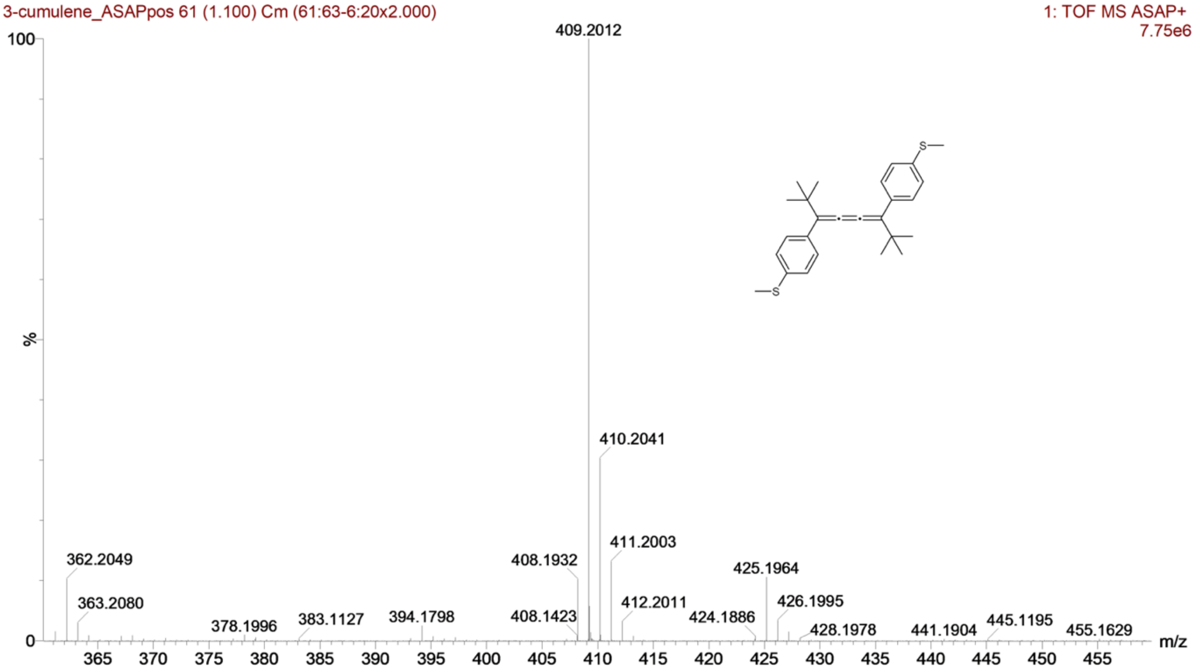

Supplementary Figure 36. HRMS spectrum of ***trans*[3]**.

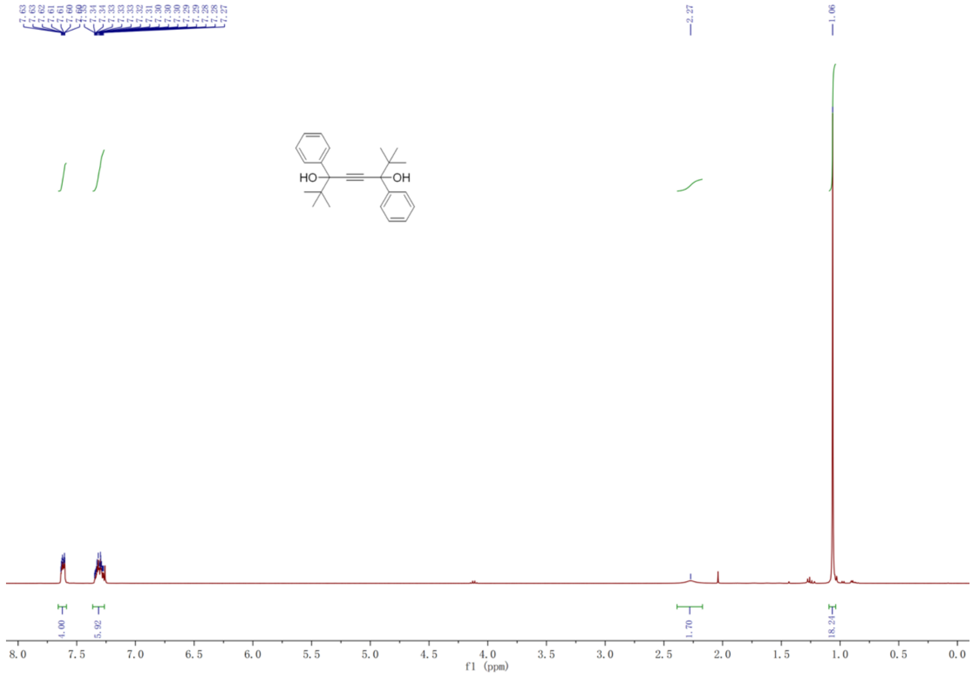

Supplementary Figure 37. ^{1}H NMR spectrum of **3**.

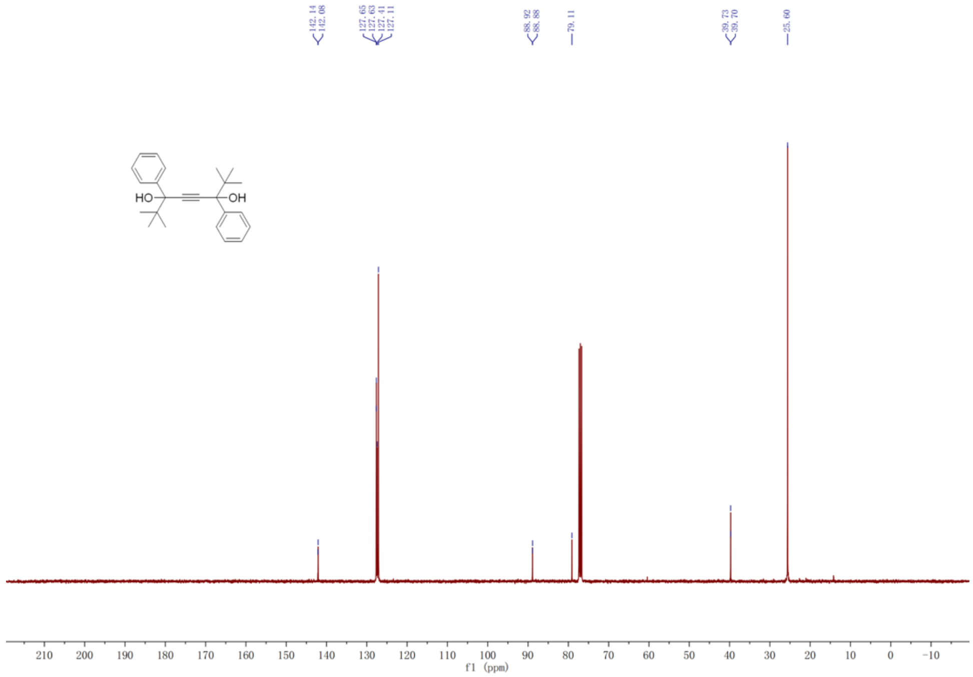

Supplementary Figure 38. ^{13}C NMR spectrum of **3**.

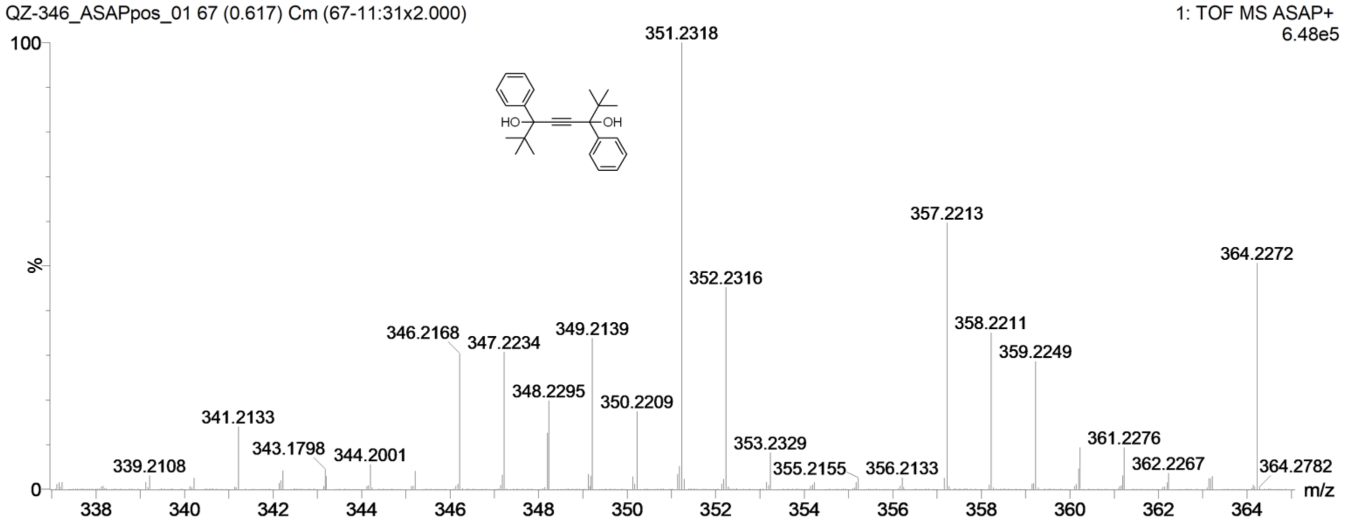

Supplementary Figure 39. HRMS spectrum of **3**.

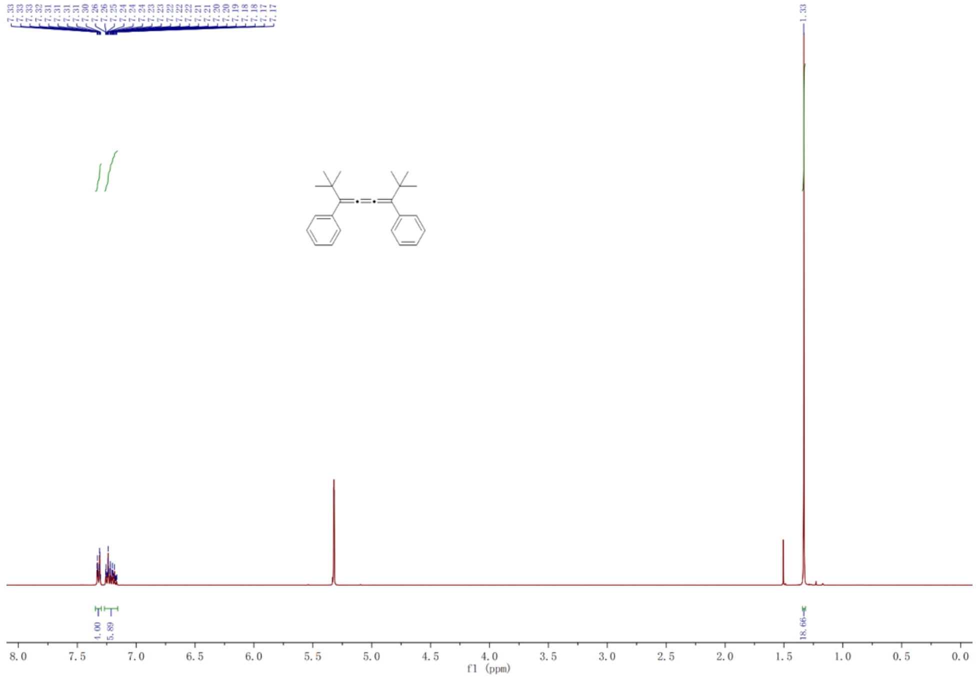

Supplementary Figure 40. ^{1}H NMR spectrum of ***cis*[3]-H.**

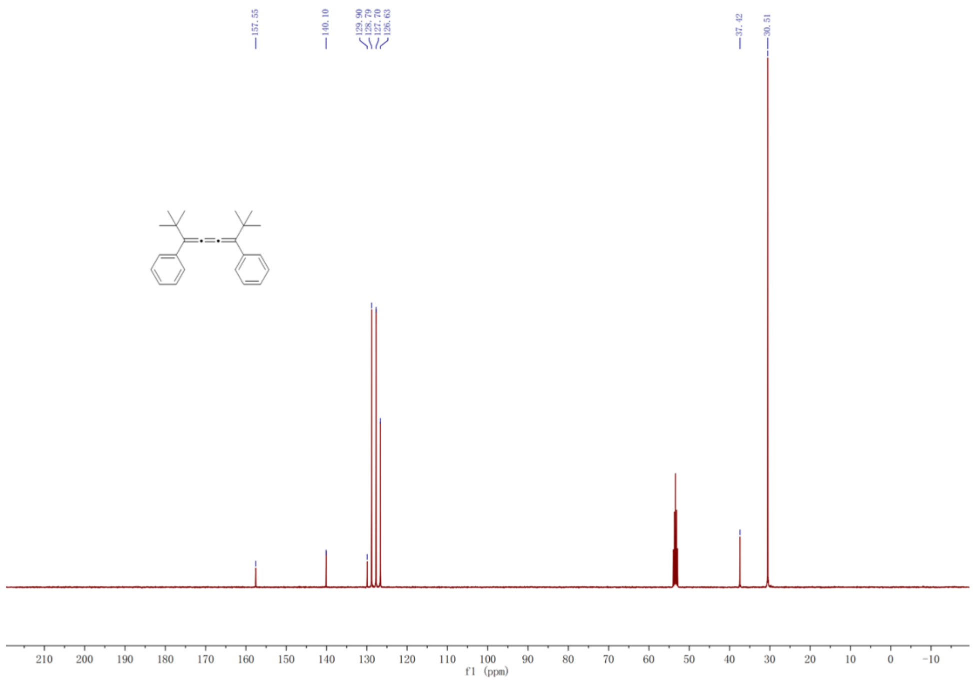

Supplementary Figure 41. ^{13}C NMR spectrum of ***cis*[3]-H.**

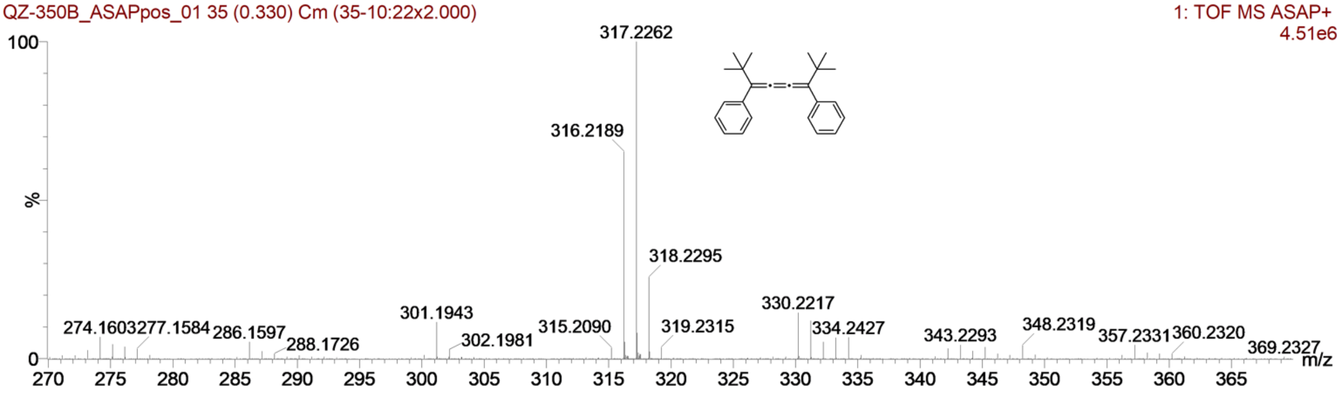

Supplementary Figure 42. HRMS of ***cis*[3]-H.**

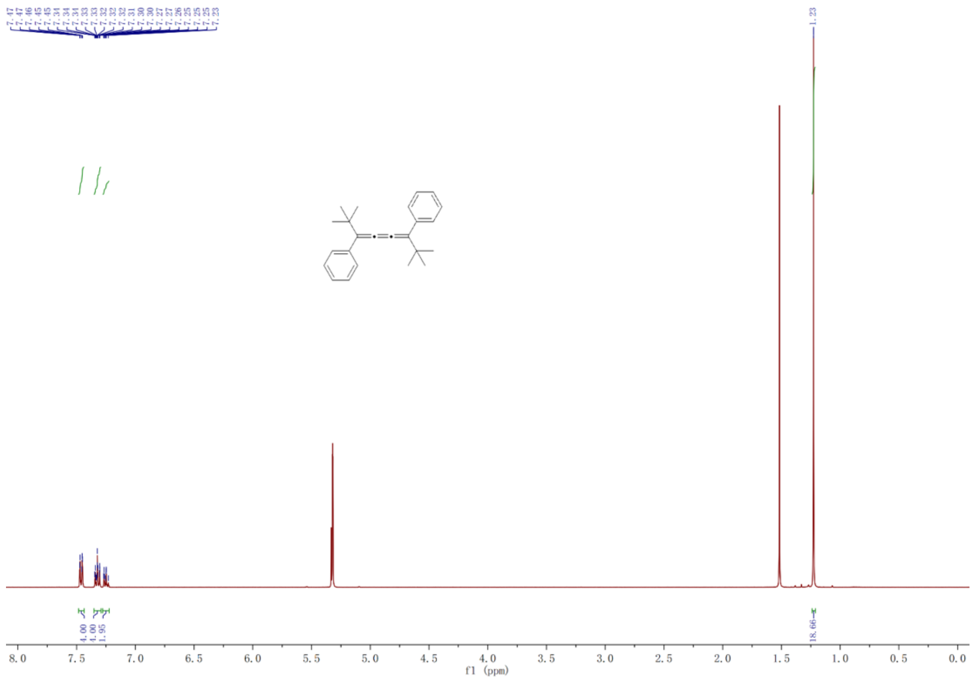

Supplementary Figure 43. ^{1}H NMR spectrum of ***trans*[3]-H.**

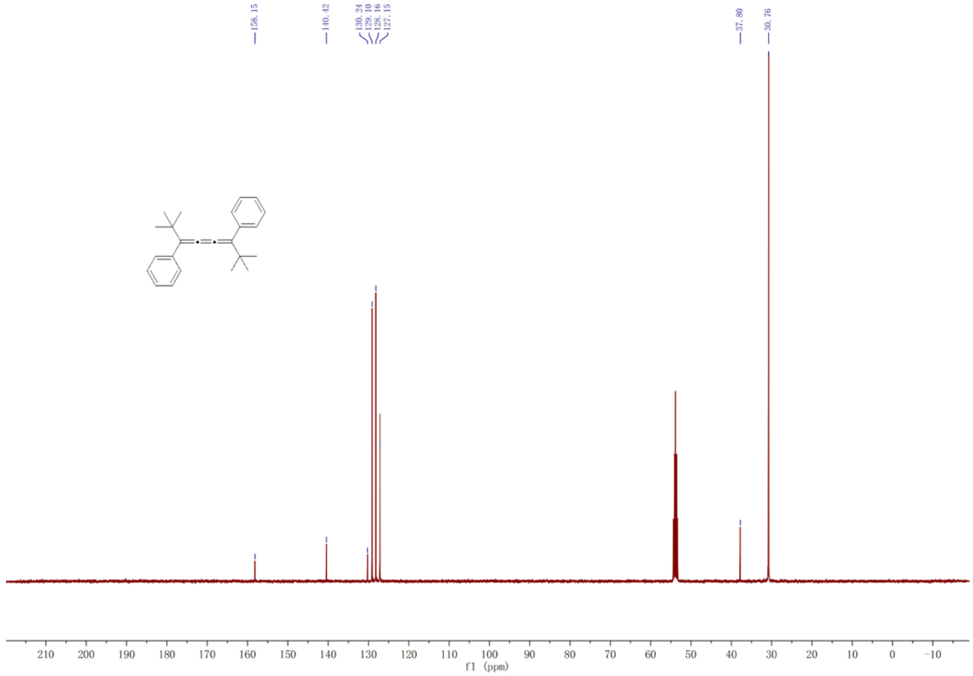

Supplementary Figure 44. ^{13}C NMR spectrum of ***trans*[3]-H.**

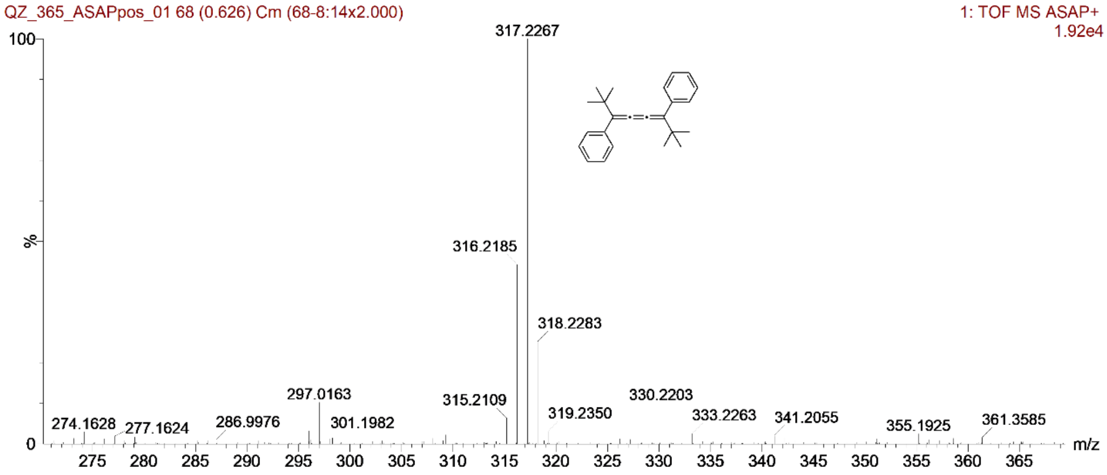

Supplementary Figure 45. HRMS of ***trans*[3]-H.**

Supplementary Table 1. Crystal data and structure determination for ***trans*[3].**

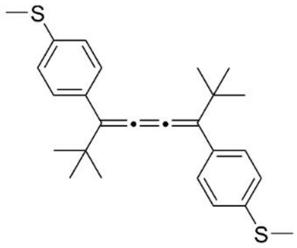

***trans*[3]**

≡

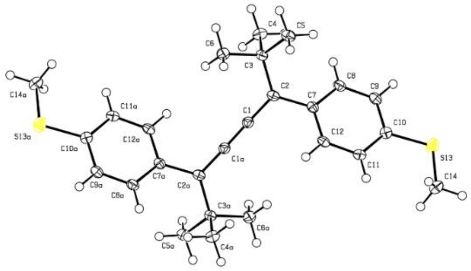

Identification code	*trans*[3]
Empirical formula	$C_{26}H_{32}S_2$
Formula mass	408.63
Temperature (K)	100 K
Crystal system	Triclinic
Space group	*P*-1 (No. 2)
λ (Å)	Mo Kα 0.71073
a (Å)	6.2296(7)
c (Å)	11.0034(19)
Z	1
V (Å^3)	565.18(16)
μ/mm^{-1}	0.245
F(000)	220
Size /mm^3	0.08 × 0.14 × 0.17
Density (g/cm^{-3})	1.201
Index ranges	$-7 \leq h \leq 8;12 \leq k \leq 11,-14 \leq l \leq 9$
Measured reflections	4411
Unique reflections	2628
Parameters	131
R_{int}	0.044
θ range (°)	3.4-29.7
R_1, wR_2	0.0534, 0.1338
S (GOF)	1.07
Max/min res. dens. (e/Å^3)	0.32/-0.34

Supplementary Table 2. Crystal data and structure determination for ***trans*[3]-H.**

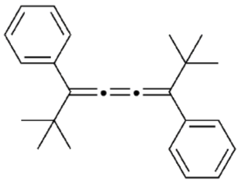

***trans*[3]-H**

≡

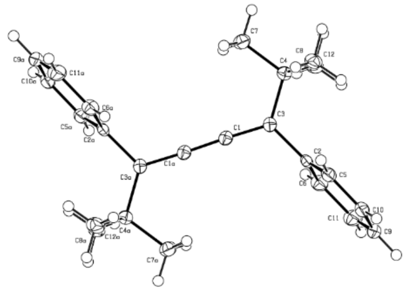

Identification code	*trans*[3]-H
Empirical formula	$C_{24}H_{28}$
Formula mass	316.46
Temperature (K)	100 K
Crystal system	Tetragonal
Space group	$P4_2/n$
λ (Å)	Cu Kα 1.54184
a (Å)	18.0677(2)
c (Å)	5.9190(1)
Z	4
V (Å^3)	1932.21(5)
μ/mm^{-1}	0.450
F(000)	688
Size /mm^3	0.20 × 0.05 × 0.05
Density (g/cm^{-3})	1.088
Index ranges	$-22 \leq h \leq 22;-22 \leq k \leq 22,-4 \leq l \leq 7$
Measured reflections	21285
Unique reflections	1938
Parameters	112
R_{int}	0.06
θ range (°)	3.5-73.0
R_1, wR_2	0.0466, 0.1229
S (GOF)	1.04
Max/min res. dens. (e/Å^3)	0.24/-0.19

Supplementary References

1 Sheldrick, G., Crystal structure refinement with shelxl. *Acta Crystallogr. Section C* **71**, 3-8 (2015).

2 Dolomanov, O.V., Bourhis, L.J., Gildea, R.J., Howard, J.A.K., & Puschmann, H., Olex2: A complete structure solution, refinement and analysis program. *J. Appl. Cryst.* **42**, 339-341 (2009).

3 Venkataraman, L. *et al.*, Single-molecule circuits with well-defined molecular conductance. *Nano Lett.* **6**, 458 - 462 (2006).

4 Nagahara, L.A., Thundat, T., & Lindsay, S.M., Preparation and characterization of stm tips for electrochemical studies. *Rev. Sci. Instrum.* **60**, 3128-3130 (1989).

5 Havu, V., Blum, V., Havu, P., & Scheffler, M., Efficient integration for all-electron electronic structure calculation using numeric basis functions. *J. Comp. Phys.* **228**, 8367-8379 (2009).

6 Blum, V. *et al.*, Ab initio molecular simulations with numeric atom-centered orbitals. *Comp. Phys. Commun.* **180**, 2175-2196 (2009).
